# Metabolomic analysis of male combat veterans with post traumatic stress disorder

**DOI:** 10.1371/journal.pone.0213839

**Published:** 2019-03-18

**Authors:** Synthia H. Mellon, F. Saverio Bersani, Daniel Lindqvist, Rasha Hammamieh, Duncan Donohue, Kelsey Dean, Marti Jett, Rachel Yehuda, Janine Flory, Victor I. Reus, Linda M. Bierer, Iouri Makotkine, Duna Abu Amara, Clare Henn Haase, Michelle Coy, Francis J. Doyle, Charles Marmar, Owen M. Wolkowitz

**Affiliations:** 1 Department of Obstetrics, Gynecology & Reproductive Sciences, University of California, San Francisco, CA, United States of America; 2 Department of Psychiatry and UCSF Weill Institute for Neurosciences, University of California, San Francisco, CA, United States of America; 3 Integrative Systems Biology, US Army Medical Research and Materiel Command, USACEHR, Fort Detrick, Frederick, MD, United States of America; 4 School of Engineering and Applied Sciences, Harvard University, Cambridge, MA, United States of America; 5 Department of Psychiatry, James J. Peters VA Medical Center, Bronx, NY and Department of Psychiatry, Icahn School of Medicine at Mount Sinai, New York, NY, United States of America; 6 Department of Psychiatry, New York University Langone Medical School, New York, NY, United States of America; 7 Stephen and Alexandra Cohen Veteran Center for Posttraumatic Stress and Traumatic Brain Injury, New York, NY, United States of America; Weill Cornell Medical College in Qatar, QATAR

## Abstract

Posttraumatic stress disorder (PTSD) is associated with impaired major domains of psychology and behavior. Individuals with PTSD also have increased co-morbidity with several serious medical conditions, including autoimmune diseases, cardiovascular disease, and diabetes, raising the possibility that systemic pathology associated with PTSD might be identified by metabolomic analysis of blood. We sought to identify metabolites that are altered in male combat veterans with PTSD. In this case-control study, we compared metabolomic profiles from age-matched male combat trauma-exposed veterans from the Iraq and Afghanistan conflicts with PTSD (n = 52) and without PTSD (n = 51) (‘Discovery group’). An additional group of 31 PTSD-positive and 31 PTSD-negative male combat-exposed veterans was used for validation of these findings (‘Test group’). Plasma metabolite profiles were measured in all subjects using ultrahigh performance liquid chromatography/tandem mass spectrometry and gas chromatography/mass spectrometry. We identified key differences between PTSD subjects and controls in pathways related to glycolysis and fatty acid uptake and metabolism in the initial ‘Discovery group’, consistent with mitochondrial alterations or dysfunction, which were also confirmed in the ‘Test group’. Other pathways related to urea cycle and amino acid metabolism were different between PTSD subjects and controls in the ‘Discovery’ but not in the smaller ‘Test’ group. These metabolic differences were not explained by comorbid major depression, body mass index, blood glucose, hemoglobin A1c, smoking, or use of analgesics, antidepressants, statins, or anti-inflammatories. These data show replicable, wide-ranging changes in the metabolic profile of combat-exposed males with PTSD, with a suggestion of mitochondrial alterations or dysfunction, that may contribute to the behavioral and somatic phenotypes associated with this disease.

## Introduction

Individuals with post-traumatic stress disorder (PTSD) have increased rates of several serious medical diseases, including cardiovascular disease, diabetes, autoimmune diseases and early mortality, suggesting widespread physical concomitants of PTSD [1]. Specific metabolic changes (“metabolic signatures” [2]) have been reported in several central nervous system disorders [3–10], childhood maltreatment [11]; exhaustion disorder [12], as well as in cardiovascular and coronary artery disease [13, 14] and insulin resistance [15–17]. Although analysis of altered metabolic pathways may provide new information about disease pathophysiology and may suggest novel drug targets, a metabolic signature has not yet been identified in PTSD, but a recent small study of non-combat, highly traumatized civilian subjects with PTSD (mean Clinician-Administered PTSD Scale, CAPS, scores >80) suggests there may be metabolomic differences in PTSD [18]. In that study of 20 PTSD and 18 control subjects, 19 metabolites were identified as being different between groups, and included several phospholipids, fatty acid metabolites, nucleosides, and bile acids, and whose abundance correlated with symptom severity [18].

Comorbidities of combat PTSD with cardiovascular disease, metabolic syndrome, and other diseases [19] suggest that there are likely to be metabolic differences between subjects with combat-related PTSD and controls. Therefore, we determined the plasma metabolomic profiles in male United States veterans from the Iraq and Afghanistan conflicts of 2001–2014 (Operation Enduring Freedom/Operation Iraqi Freedom, OEF/OIF) who endured combat-related trauma and developed PTSD, and compared these profiles to those in veterans who also endured combat-related trauma but did not develop PTSD. We also determined if these metabolites are associated with severity of PTSD symptoms. In order to lessen the likelihood of false positive results, which is attendant upon examining a large number of metabolites, we tested two separate cohorts of such subjects, to see which metabolite differences were replicable.

Animal models of PTSD have suggested disrupted brain energy metabolism [20], and studies of prolonged stress in mice have found alterations in mitochondrial pathways that were associated with hippocampal and amygdala apoptosis [21–23]. Further, studies of human blood from the cohort used in the current study [24] and other studies of blood and postmortem brain [25, 26] have shown several differentially methylated or dysregulated genes associated with mitochondrial function in PTSD, of which 20% correlated significantly with the severity of PTSD symptoms [26]. In addition, a study of childhood maltreatment [27] and major depression [28], which may share some behavioral features with PTSD, have also demonstrated significant alterations in mitochondrial function, which may be related to both increased inflammation and increased oxidative stress. Therefore, we hypothesized that subjects with PTSD would have, among other things, differences in metabolites that reflect mitochondrial function. Our data identify a metabolomic profile of combat trauma-exposed veterans with PTSD that is associated with, and may contribute to, the clinical phenotype of this disease.

## Materials and methods

### Subject recruitment

This study was approved by Institutional Review Boards of the University of California, San Francisco (San Francisco, CA), Ichan School of Medicine at Mt. Sinai (New York, NY), James J. Peters Veterans Administration Medical Center (Bronx, NY), and the New York University Langone School of Medicine (New York, NY). All participants gave written informed consent to participate in the study. This study is part of a more global Department of Defense-sponsored systems biology approach to understanding PTSD [29].

Male combat-exposed veterans from OEF/OIF were recruited at The New York University Langone School of Medicine and Mt. Sinai/James J. Peters VA Medical Center through flyers, presentations, newspaper and television advertisement, internet postings, and referral from clinicians. PTSD was diagnosed with structured clinical interviews. Inclusion criteria for PTSD-positive subjects included current war-zone trauma-related PTSD of at least 3 months duration and having Clinician Administered PTSD Scale (CAPS) [30] scores >40, while control (combat trauma-exposed PTSD-negative) veterans had no lifetime history of PTSD and had CAPS scores <20. All subjects were between 20 and 60 years old, and had English as their primary language. Exclusion criteria included: 1) history of alcohol dependence within the past 8 months; 2) history of drug abuse or dependence within the last 12 months; 3) lifetime history of any psychiatric disorder with psychotic features, bipolar or obsessive-compulsive disorder; 4) current exposure to recurrent trauma or a traumatic event within the past 3 months; 5) prominent suicidal or homicidal ideation; 6) neurologic disorder or systemic illness affecting central nervous system function; 7) clinical history of anemia or blood donation within the past 2 months; 8) changes in the past two months of psychotropic medication, anticonvulsants, antihypertensive medication, sympathomimetic medication, medications associated with neurogenesis or systemic steroid medication; 9) diagnosis of moderate or severe traumatic brain injury (TBI) on the Ohio State University TBI Identification Method-Short Form [31]; or 10) classification of mild TBI with a score ≥ 8 for current symptoms on the post-concussive symptom checklist.

### Subjects and samples

Combat-exposed male OEF/OIF veterans with PTSD (n = 52) or combat-exposed, male OEF/OIF veterans without PTSD (n = 51) (“Discovery group”) were matched by age. Of the PTSD-positive subjects, 27 were also diagnosed with concurrent Major Depressive Disorder (MDD), as assessed by the Structured Clinical Interview for DSM-IV (SCID) [32]. Depression symptom severity was assessed with the self-rated Beck Depression Inventory-II (BDI-II) [33]. Following urinary toxicology screening for cannabinoids, cocaine, barbiturates, benzodiazepine, opiates, methadone, amphetamines, and phencyclidine, blood was collected for complete blood count, electrolytes, glucose, urea nitrogen, creatinine, glycated hemoglobin (HbA1c) and the liver function tests as specified by a CLIA certified clinical laboratory. Subjects reported to the laboratory at 7:30 AM having fasted overnight. Vital signs, weight, height and waist-to-hip ratio were measured. Following a period of rest, blood was drawn at 8:00 AM. Blood for metabolomics assays was collected into tubes containing EDTA, which were inverted 8–10 times before being placed on ice for up to 30 minutes, centrifuged at 4°C for 15 minutes at 1100 x g, following which plasma was removed and stored in 500 μL aliquots at -80°C until processed.

A second group of combat trauma-exposed male OEF/OIF veterans with PTSD (n = 31) and combat trauma-exposed, male OEF/OIF veterans without PTSD (n = 31) was enrolled as a “Test group” and followed the identical protocol as the initial Discovery group.

### Sample preparation and metabolic profiling

Metabolomic profiling of all plasma samples was performed at Metabolon, Inc. (Durham, NC), as described [34–36], with plasma from both PTSD positive and PTSD negative subjects being run in the same batches. Details of the analytic procedures are provided in S1 File and metabolomic data are found in S1 Dataset.

### Statistical analysis

For statistical analyses and data display purposes, values below the limits of detection were replaced with the compound minimum (minimum value imputation). All metabolomic data were transformed in the same manner using the Blom transformation [37]. The Blom transformation is a rank-based normalization transformation, essentially a non-parametric procedure used to lessen deviations from the assumption of normality. As such, it does not distort the underlying data more than any other non-parametric transformation. Statistical analyses of Blom-transformed data were performed using SPSS (IBM, Armonk, NY) and R (http://cran.r-project.org). All tests were 2-tailed with an alpha = 0.05. Significance values between 0.05 and 0.1 are reported as trends. Multiple comparisons were accounted for by estimating the false discovery rate using q-values [38]. Q-values are p-values adjusted for the false discovery rate. They indicate the percent of significant results that will result in false positives, rather than the percent of all tests that will result in false positives; this usually results in smaller numbers of false positives. While a higher *q*-value indicates diminished confidence, it does not necessarily rule out the significance of a result. Other lines of evidence may be taken into consideration when determining whether a result merits further scrutiny. Such evidence may include a) inclusion in a common pathway that includes a strategically significant compound or b) residing in a similar functional biochemical family with other significant compounds. In addition, bootstrapping based upon random sampling of the subjects was used as an additional statistical method to validate the findings across the Discovery and Test groups. Biochemical differences between groups were assessed by *t*-tests, analyses of covariance (ANCOVA) and correlation analyses. Independent samples t-test for continuous variables and chi-square test for dichotomous variables were used to examine participants’ baseline between-group differences.

## Results

### Demographics in the “Discovery group”

There were no significant differences in age, ethnicity between the 52 PTSD subjects and 51 controls in the “Discovery group” (Table 1). There were no significant differences in HbA1c, cholesterol, HDL, LDL, triglycerides, waist-to-hip ratios, sodium, calcium, CO_2_, blood urea nitrogen or liver function tests. PTSD-positive subjects had increased body mass index (BMI) (p<0.05), high sensitivity C-reactive protein (p<0.05), glucose (p<0.01), insulin (p<0.01), homeostatic model assessment-estimated insulin resistance (HOMA-IR) (p<0.02), creatinine (p<0.01), pulse rate (p<0.01), hemoglobin (p<0.02), and hematocrit (p<0.03), compared to the PTSD-negative subjects (Table 1). Although the PTSD-positive group had a higher average BMI than the PTSD-negative group, their waist-to-hip ratio, which may be a better predictor of health [39], was not significantly different. In addition to the differences in CAPS scores that were used for inclusion/exclusion, BDI scores were significantly higher in PTSD subjects (p<0.001), among whom 52% (29/52) (chi-square) also met criteria for MDD and 29% (15/52) were receiving antidepressant medications (chi-square). The PTSD group took more medications than did the control group. In the Discovery group, 40.4% (21/52) of the PTSD group and 17.6% (9/51) of the control group took some type of medication (chi-square p<0.01). There were no significant between-group differences in the prevalence of hypertension, heart attacks, stable angina, stroke, or diabetes, or in the number of subjects taking statins, non-steroidal anti-inflammatories, analgesics or oral hypoglycemic agents.

**Table 1:** Demographic and clinical characteristics of combat veterans with PTSD and controls in Discovery and Test groups.

	Discovery Group					Test Group				
	PTSD - N: 51	PTSD + N: 52	Statistic		Χ^2^ (p)	PTSD - N: 31	PTSD + N: 31	Statistic		Χ^2^ (p)
	Means± SD	Means± SD	T	P		Means± SD	Means± SD	T	P	
**Sociodemographi*c***										
Age (years, mean ± SD)	33.69 ± 9.03	34.02 ± 8.69	0.19	0.85		30.61 ±5.66	31.23 ± 5.45	0.43	0.66	
Years of education (mean ± SD)	14.74 ± 2.33	13.75 ± 1.80	2.42	0.02		14.84 ± 2.34	13.90 ± 2.16	1.63	0.11	
Gender	All males	All males				All males	All males			
Smokers (n)	3	11			0.02*	1	0			0.17
Hispanic/ Non-Hispanic (n)	20/31	26/26			0.27	6/25	12/19			0.09
**Metabolic measurements**	**Means± SD**	**Means± SD**	**T**	**P**	**Χ**^**2**^	**Means± SD**	**Means± SD**	**T**	**P**	**Χ**^**2**^
BMI	28.24 ± 4.15	30.03 ± 5.12	1.94	0.05*		28.78 ± 5.74	30.00 ± 5.00	0.89	0.373	
HbA1c	5.48 ± 0.42	5.35 ± 0.91	0.85	0.39		5.18 ± 0.42	5.52 ± 0.87	1.95	0.05*	
Cholesterol (mg/dl)	171.23 ± 26.54	175.75 ± 35.07	0.74	0.46		171.55 ± 29.30	185.58 ± 36.71	1.66	0.10	
CRP	1.65 ± 2.30	3.38 ±5.64	2.01	0.05*		1.56 ± 2.54	4.09 ± 5.17	2.45	0.02*	
Glucose (mg/dL)	79.94 ± 13.76	91.42 ± 23.57	3.01	<0.01*		82.19 ± 9.19	88.26 ± 25.99	1.22	0.22	
Insulin (microunits/mL)	12.15 ± 10.43	19.36 ±18.84	2.60	0.01*		12.79 ± 10.07	18.31 ± 15.94	1.63	0.11	
HOMA-IR	2.64 ± 3.40	4.65 ± 4.70	2.47	0.02*		2.74 ± 2.46	4.68 ± 6.36	1.58	0.12	
HDL (mg/dL)	48.09 ± 13.51	46.31 ± 11.11	0.73	0.47		52.58 ± 12.12	49.48 ± 14.29	0.92	0.36	
LDL (mg/dL)	102.93 ± 24.28	104.18 ± 30.18	0.23	0.82		96.66 ± 26.36	111.25 ± 37.60	1.77	0.08	
Triglycerides (mg/dL)	107.74 ± 110.36	123.25 ± 63.41	0.87	0.38		113.38 ± 83.92	124.13 ± 77.80	0.52	0.60	
Waist to hip ratio	0.89 ± 0.12	0.91 ± 0.08	0.96	0.34		0.87 ± 0.19	0.86 ± 0.25	0.22	0.83	
Sodium (mEq/L)	140.20 ± 1.43	140.19 ± 1.81	0.01	0.99		140.48 ± 1.48	140.58 ± 1.84	0.23	0.82	
Calcium (mg/dL)	9.24 ± 0.43	9.26 ± 0.38	0.23	0.82		9.40 ± 0.39	9.23 ± 0.32	1.86	0.07	
Chloride (mEq/L)	103.24 ± 2.34	104.35 ± 2.84	2.16	0.03*		103.10 ± 2.38	103.87 ± 3.38	1.04	0.30	
Potassium (mEq/L)	3.93 ± 0.35	4.16 ± 0.35	3.24	<0.01*		4.01 ± 0.42	4.10 ± 0.41	0.86	0.39	
Total Protein (g/dL)	6.85 ± 0.44	7.26 ± 0.46	4.56	<0.01*		7.22 ± 0.48	7.32 ± 0.48	0.81	0.42	
Albumin (g/dL)	4.24 ± 0.30	4.45 ± 0.31	3.39	0.01*		4.57 ± 0.26	4.56 ± 0.23	0.67	0.88	
Alkaline phosphatase (U/L)	61.45 ± 18.31	69.38 ± 18.68	2.17	0.03*		67.32 ± 22.96	69.68 ± 20.59	0.42	0.67	
Aspartate transaminase (U/L)	26.25 ± 14.26	30.04 ± 16.61	1.24	0.22		30.97 ± 24.29	33.19 ± 17.13	0.42	0.67	
Alanine transaminase (U/L)	29.55 ± 17.46	36.60 ± 23.22	1.74	0.08		34.39 ± 30.31	37.13 ± 23.82	0.39	0.69	
Gamma-glutamyl transferase (GGT) (U/L)	27.17 ± 25.02	35.35 ± 24.61	1.66	0.09		53.32 ± 142.58	35.72 ± 17.11	0.66	0.51	
Blood Urea Nitrogen (mg/dL)	15.33 ± 3.85	14.00 ± 3.82	1.76	0.08		14.68 ± 2.75	15.42 ± 3.20	0.98	0.33	
Creatinine (mg/dL)	1.04 ± 0.19	0.94 ± 0.17	2.89	<0.01*		1.03 ± 0.16	1.04 ± 0.19	0.30	0.76	
CO2 (mEq/L)	27.61 ± 1.88	27.56 ± 2.54	0.11	0.91		27.02 ± 2.51	27.30 ± 2.53	0.44	0.66	
Pulse (beats/min)	64.20 ± 10.65	72.35 ± 9.91	4.02	<0.01*		65.48 ± 12.22	73.16 ±11.15	2.58	0.01*	
Hemoglobin (g/dL)	14.22 ± 1.06	14.74 ± 1.21	2.29	0.02*		14.58 ±0.67	14.84 ± 1.18	1.07	0.29	
Hematocrit (%)	41.98 ± 2.85	43.46 ± 3.33	2.42	0.02*		42.88 ± 2.02	43.99 ± 3.23	1.62	0.11	
**Medications**	**Means± SD**	**Means± SD**	**T** ^***a***^	**P**	**Χ**^**2**^ **(p)**	**Means± SD**	**Means± SD**	**T** ^***a***^	**P**	**Χ**^**2**^ **(p)**
Taking sedatives (n)	1	6			0.05*	3	4			0.51
Taking statins (n)	1	2			0.57	0	2			0.11
Taking antidepressants (n)	2	15			<0.01*	2	5			0.14
Taking anticonvulsants (n)	0	3			0.08	0	2			0.11
Taking anti-inflammatories (n)	5	4			0.70	0	1			0.27
Taking anti-diabetic medication (n)	1	1			0.99	1	1			0.89
Taking antibiotics (n)	1	1			0.99	0	0			
Taking beta-blockers (n)	1	1			0.99	0	0			
Taking any medication (n)	9	21			<0.01	5	10			0.10
**Comorbid Diseases**	**Means± SD**	**Means± SD**	**T**	**P**	**Χ**^**2**^ **(p)**	**Means± SD**	**Means± SD**	**T**	**P**	**Χ**^**2**^ **(p)**
Clinical hypertension (n)	4	9			0.08	3	6			0.52
Heart attack ^*a*^ (n)	1	0			0.31	0	1			0.30
Stable angina (n)	1	2			0.50	0	1			0.30
Diabetes (n)	1	3			0.36	1	1			0.98
**Clinical Measures**	**Means± SD**	**Means± SD**	**T**	**P**	**Χ**^**2**^ **(p)**	**Means± SD**	**Means± SD**	**T**	**P**	**Χ**^**2**^ **(p)**
CAPS total current	2.90 ± 4.24	68.02 ± 16.80	26.85	<0.01*		5.23 ± 6.20	71.77 ± 17.11	20.36	<0.01*	
CAPS total lifetime	8.65 ± 7.83	90.87 ± 15.47	33.93	<0.01*		10.39 ± 9.46	92.71 ± 16.54	24.06	<0.01*	
Concurrent MDD diagnosis (n)	n = 0	n = 27			<0.01*	n = 0	n = 20			<0.01*

^**a**^ Myocardial infarction, coronary occlusion or coronary thrombosis

* p ≤ 0.05

### Demographics in the “Test group”

In the “Test group” of 31 PTSD-negative and 31 PTSD-positive subjects, demographic and clinical characteristics were similar to the Discovery group, except there were no statistically significant differences in BMI, glucose, insulin, HOMA-IR, creatinine, hemoglobin, or hematocrit between the PTSD-positive and -negative groups. Like the Discovery group, the Test group PTSD-positive subjects had increased high sensitivity C-reactive protein (p<0.02) and pulse rate (p<0.01) (Table 1). In addition to the differences in CAPS scores that were used for inclusion/exclusion, BDI scores were significantly higher in PTSD subjects (p<0.001) in the test group, among whom 64.5% (chi-square) also met criteria for MDD and 16.1% were receiving antidepressant medications (chi-square), and 32.2% of the PTSD subjects and 16.1% of the control subjects took some type of medication (chi-square p = 0.1). There were no significant between-group differences in the prevalence of other comorbid diseases.

### Metabolite differences in PTSD-positive vs PTSD-negative subjects in both the Discovery and Test groups

The analysis of metabolites in plasma of the Discovery and Test groups included all detectable compounds of known identity. Of 4400 metabolites and xenobiotics potentially identifiable by our mass spectrometry platforms, 370 named compounds were identified in plasma of our initial Discovery group and 623 named compounds were identified in plasma of our validating Test group. These differences arose because the two metabolomic analyses used slightly different methodologies that resulted in detection and identification of some different compounds in each analysis. Hence, only 244 compounds that were identified in both the Discovery and Test samples were used in the analyses (Table A in S1 File, S1 Dataset, and Tables 2 and 3). Summaries of the numbers of compounds that achieved statistical significance (*p*≤0.05) are shown in Tables 2 and 3.

**Table 2:** Biochemicals that achieved statistical significance (p<0.05) between groups of male combat veterans with and without PTSD in the Discovery group.

			Discovery Group											
Super Pathway	Sub Pathway	Biochemical Name	Control		PTSD		PTSD/ Control	p-value^a^	Cohen's d	q-value^b^	ANCOVA, covarying for:			
											BMI	HbA1C	Glucose	Cotinine
			Mean	SD	Mean	SD								
Amino acid	Glutamate metabolism	glutamine	1.050	0.145	0.978	0.133	**0.932**	**0.010**	0.592	0.241	.010	.014	.004	.012
	Histidine metabolism	trans-urocanate	1.051	0.539	0.834	0.427	**0.794**	**0.033**	0.460	0.378	.041	.030	.100	.018
	Phenylalanine & tyrosine metabolism	phenyllactate (PLA)	0.781	0.327	1.033	0.639	**1.323**	**0.026**	0.491	0.359	.071	.049	.126	.025
	Urea cycle; arginine-, proline-, metabolism	arginine	1.142	0.297	1.013	0.374	**0.887**	**0.031**	0.386	0.378	.083	.007	.069	.046
	Leucine, Isoleucine and Valine Metabolism	3-hydroxyisobutyrate	1.094	0.301	0.987	0.296	**0.902**	**0.040**	0.333	0.378	.035	.077	.038	.051
	Glutathione metabolism	5-oxoproline	0.943	0.149	1.073	0.187	**1.138**	**<0.001**	0.759	0.000	.000	.000	.000	.001
Carbohydrate	Glycolysis, gluconeogenesis, pyruvate metabolism	pyruvate	1.056	0.736	1.272	0.649	**1.205**	**0.017**	0.302	0.274	.050	.017	.056	.025
		lactate	0.937	0.283	1.239	0.341	**1.322**	**<0.001**	0.963	0.000	.000	.000	.000	.000
Energy	Krebs cycle	citrate	1.071	0.267	0.948	0.231	**0.885**	**0.017**	0.478	0.274	.022	.037	.013	.024
Lipid	Essential fatty acid	linolenate [alpha or gamma; (18:3n3 or 6)]	1.198	0.533	1.013	0.577	**0.846**	**0.039**	0.342	0.378	.018	.051	.049	.039
		dihomo-linoleate (20:2n6)	1.196	0.598	0.979	0.510	**0.819**	**0.042**	0.395	0.378	.021	.053	.053	.036
		dihomo-linolenate (20:3n3 or n6)	1.096	0.407	0.951	0.393	**0.868**	**0.039**	0.375	0.378	.014	.077	.035	.035
		docosahexaenoate (DHA; 22:6n3)	1.117	0.568	0.938	0.501	**0.840**	**0.047**	0.317	0.378	.032	.026	.046	.092
	Long chain fatty acid	10-nonadecenoate (19:1n9)	1.212	0.525	1.041	0.631	**0.859**	**0.047**	0.275	0.378	.023	.078	.039	.029
		eicosenoate (20:1n9 or 11)	1.282	0.646	1.028	0.625	**0.802**	**0.019**	0.391	0.282	.014	.033	.026	.019
	Sphingolipid	sphingosine-1-phosphate	0.947	0.386	1.122	0.459	**1.185**	**0.046**	0.399	0.378	.076	.056	.029	.061
	Carnitine metabolism	octanoylcarnitine	0.936	0.406	1.241	0.690	**1.326**	**0.004**	0.529	0.129	.005	.012	.010	.001
		hexanoylcarnitine	1.003	0.288	1.174	0.439	**1.171**	**0.047**	0.456	0.378	.100	.090	.071	.030
		decanoylcarnitine	0.970	0.432	1.272	0.643	**1.311**	**0.003**	0.550	0.116	.003	.010	.006	.001
	Sterol/Steroid	cortisol	0.912	0.398	1.085	0.339	**1.190**	**0.012**	0.485	0.251	.010	.008	.012	.003
Nucleotide	Purine metabolism, (hypo)xanthine/inosine containing	hypoxanthine	0.943	0.384	1.286	0.687	**1.364**	**0.013**	0.628	0.251	.040	.026	.011	.052
		GABR	0.575	0.208	0.475	0.170	**0.826**	**0.009**	0.523	0.241	.018	.004	.014	.009
		GLYCOLITIC RATIO	1.977	1.154	2.787	1.145	**1.410**	**<0.001**	0.619	0.000	.000	.000	.000	.000

^a^ uncorrected p values

^b^, q values are p values adjusted for the false discovery rate

Green boxes indicate metabolites are lower in the PTSD positive group vs PTSD negative group. Red boxes indicate metabolites are higher in the PTSD positive group vs PTSD negative group

**Table 3:** Test group validation of biochemicals that achieved statistical significance (p<0.05) between groups of male combat veterans with and without PTSD in the Discovery group.

			Test Group							
Super Pathway	Sub Pathway	Biochemical Name	Control		PTSD		PTSD/ Control	p-value^a^	Cohen's d	q-value^b^
			Mean	SD	Mean	SD				
Amino acid	Glutamate metabolism	glutamine	1.050	0.120	1.020	0.124	0.971	0.353	0.250	0.455
	Histidine metabolism	trans-urocanate	1.178	0.508	1.390	2.329	1.180	0.146	0.125	0.339
	Phenylalanine & tyrosine metabolism	phenyllactate (PLA)	1.007	0.485	1.100	0.445	1.092	0.334	0.213	0.453
	Urea cycle; arginine-, proline-, metabolism	arginine	1.107	0.244	1.090	0.197	0.985	0.953	0.091	0.687
	Leucine, Isoleucine and Valine Metabolism	3-hydroxyisobutyrate	1.021	0.380	1.130	0.656	1.107	0.777	0.204	0.652
	Glutathione metabolism	5-oxoproline	0.916	0.137	0.927	0.134	1.013	0.689	0.074	0.624
Carbohydrate	Glycolysis, gluconeogenesis, pyruvate metabolism	pyruvate	1.409	0.838	2.177	1.248	**1.546**	**0.010**	0.723	0.101
		lactate	1.045	0.316	1.369	0.494	**1.311**	**0.003**	0.773	0.047
Energy	Krebs cycle	citrate	1.066	0.231	1.048	0.220	0.983	0.669	0.089	0.624
Lipid	Essential fatty acid	linolenate [alpha or gamma; (18:3n3 or 6)]	1.420	0.647	1.088	0.501	**0.766**	**0.026**	0.569	0.151
		dihomo-linoleate (20:2n6)	1.364	0.583	1.102	0.459	**0.808**	**0.066**	0.497	0.243
		dihomo-linolenate (20:3n3 or n6)	1.146	0.383	0.864	0.364	**0.753**	**0.001**	0.756	0.025
		docosahexaenoate (DHA; 22:6n3)	1.331	0.839	0.829	0.299	**0.623**	**<0.001**	0.793	0.000
	Long chain fatty acid	10-nonadecenoate (19:1n9)	1.355	0.534	1.142	0.457	**0.843**	**0.091**	0.443	0.271
		eicosenoate (20:1n9 or 11)	1.647	1.034	1.135	0.505	**0.690**	**0.014**	0.630	0.121
	Sphingolipid	sphingosine-1-phosphate	1.137	0.555	1.274	0.556	1.120	0.187	0.232	0.363
	Carnitine metabolism	octanoylcarnitine	1.831	2.794	1.594	1.439	0.871	0.832	0.108	0.660
		hexanoylcarnitine	1.553	1.245	1.504	0.905	0.968	0.841	0.046	0.660
		decanoylcarnitine	1.988	3.216	1.687	1.454	0.849	0.953	0.120	0.687
	Sterol/Steroid	cortisol	1.239	0.434	1.224	0.426	0.988	0.999	0.047	0.702
Nucleotide	Purine metabolism, (hypo)xanthine/inosine containing	hypoxanthine	1.392	0.784	1.886	0.857	**1.354**	**0.016**	0.597	0.131
		GABR	0.570	0.127	0.525	0.148	0.920	0.163	0.285	0.363
		GLYCOLITIC RATIO	2.465	1.381	3.482	1.576	**1.413**	**0.005**	0.681	0.072

^a^ uncorrected p values

^b^, q values are p values adjusted for the false discovery rate

Green boxes indicate metabolites are lower in the PTSD positive group vs PTSD negative group. Red boxes indicate metabolites are higher in the PTSD positive group vs PTSD negative group. Light green boxes in the Test group indicate metabolites are lower in the PTSD positive group vs PTSD negative group at the trend level (p<0.1)

All of the significant between-group differences reported below were also confirmed by bootstrap analyses (Figure A in S1 File). The information from the metabolic profiling identified significant differences between PTSD-positive and -negative subjects in biochemical pathways involved in glucose metabolism, energy utilization and lipid metabolism in both the Discovery and Test groups.

#### Carbohydrates

Carbohydrates, amino acids and fats can be used to generate reducing equivalents (NADH) and ATP. The main, highly significant finding of our study was that lactate (Discovery group: p<1.6x10^-6^, q = 0.00; Test group: p = 0.003; q = 0.047) and pyruvate (Discovery group: p = 0.017, q = 0.27; Test group: p = 0.010, q = 0.10), two products of anaerobic respiration in glycolysis, were significantly elevated in the PTSD-positive subjects compared to the PTSD negative subjects. Among the intermediates in the TCA cycle (aerobic respiration), citrate was decreased in PTSD subjects in the Discovery group (p = 0.017, q = 0.27), although this finding was not replicated in the Test group (p = 0.669, q = 0.62). To assess the relative contribution of metabolites from anaerobic and aerobic respiration in our subjects, we defined a ratio indicative of anaerobic relative to aerobic respiration, and calculated a ratio from the relative amounts of pyruvate, lactate and citrate in each subject’s plasma ([Pyruvate + Lactate]/Citrate), and called this the “glycolytic ratio”. The glycolytic ratio was significantly higher in veterans with PTSD than in veterans without PTSD in both the Discovery (p<1x10^-4^ q = 0.00) and Test (p = 0.005; q = 0.07) groups, suggesting increased anaerobic and decreased aerobic respiration in these subjects, which may indicate mitochondrial (TCA cycle) alterations or dysfunction.

#### Lipids

Many fatty acids were lower in PTSD-positive subjects. The long chain fatty acids eicosenoate (20:1n9 or 11) (Discovery group: p = 0.019, q = 0.28; Test group: p = 0.014, q = 0.12) and 10-nonadecenoate (19:1n9) (Discovery group: p = 0.047, q = 0.38; Test group: p = 0.091, q = 0.27) were less abundant in PTSD than in the controls. These differences could result from decreased breakdown of storage lipids, increased fatty acid catabolism via ß-oxidation, and/or decreased uptake of dietary fat. Several long-chain essential fatty acids that must be absorbed from the diet, including linolenate (18:3n3 or 6) (Discovery group: p = 0.039, q = 0.38; Test group: p = 0.026, q = 0.15), dihomo-linoleate (20:2n6) (Discovery group: p = 0.042, q = 0.38; Test group: p = 0.066, q = 0.24), and dihomo-linolenate (20:3n3 or n6) (Discovery group: p = 0.039, q = 0.38; Test group: p = 0.001, q = 0.02), and docosahexaenoate (DHA, 22:6n3) (Discovery group: p = 0.047, q = 0.38; Test group: p<0.001, q = 0.00) were also decreased in PTSD versus controls. These data suggest that decreased dietary fat uptake, mitochondrial alterations or dysfunction, or differences in the gut microbiome may contribute to the observed decrease in free fatty acids in PTSD.

#### Hypoxanthine

Levels of hypoxanthine were higher in PTSD compared to controls (Discovery group: p = 0.013, q = 0.25; Test group: p = 0.016, q = 0.13). Hypoxanthine is a naturally occurring purine derivative that is involved in ATP catabolism and the salvage pathway for purine synthesis. However, we did not see elevated levels of xanthine and uric acid, other products of the purine catabolism pathway. Increased levels of hypoxanthine, a substrate for xanthine oxidase, may result in generating reactive oxygen species (ROS) [40].

#### Other metabolites

Information about metabolites that were significantly different only in the Discovery group, but not the smaller Test group, can be found in Tables 2 and 3 and in Supplementary Material (Table A in S1 File and S1 Dataset).

### ANCOVA analyses with meta-data in the Discovery group

**Hemoglobin A1c (HbA1c)** reflects average blood glucose levels over the previous three months. Because our data suggest that metabolite differences between PTSD and control subjects may reflect differences in use of energy sources (glucose vs fatty acids), we evaluated status of glucose dysregulation as a covariate. Only 2 subjects (1 PTSD, 1 control) in the Discovery group carried pre-existing diagnoses of diabetes, and HbA1c values were not different between groups (Table 1). Using Hb1Ac as a covariate had little effect on metabolite differences between PTSD-positive and–negative groups (Table 2): 14 of 21 metabolites that were significantly different between PTSD-positive and -negative subjects remained significant, while the other 7 metabolites were now trends (p<0.09). Similarly, using glucose as a covariate had little effect on differences in metabolite concentrations between PTSD-positive and–negative subjects.

#### BMI

Because we identified potential differences in energy metabolism between PTSD and control subjects, we tested BMI as covariate. Although BMI was significantly greater in PTSD-positive than in PTSD-negative subjects in the Discovery group (Table 1 and [41]), using BMI as a covariate had little effect on differences in metabolite concentrations between PTSD-positive and–negative subjects (Table 2). After covarying for BMI, 17 of the 21 metabolites that were significantly different between groups remained significantly different, and the other 4 now trended toward significance (p<0.083).

Because high BMI may be co-morbid with PTSD in the Discovery group, and because there were no differences in BMI between PTSD and control subjects in the Test group, we also covaried for BMI in the Test group to see if any of the metabolites were influenced by BMI. The metabolites identified as statistically significant between PTSD and control subjects remained significant after covarying for BMI (all p<0.035).

In addition, we also covaried for waist-to-hip ratio instead of BMI, since it may be a better predictor of health [39], and we found that none of our main findings were altered. These analyses suggest that our findings are not driven by anthropometric variables.

#### Cotinine

To test the possible impact of tobacco use on the findings, we evaluated plasma cotinine levels as a covariate, since cotinine is the predominant metabolite of nicotine. Using cotinine as a covariate had little effect on metabolite differences between PTSD-positive and–negative groups (Table 2): 17 of 21 metabolites that were significantly different between PTSD-positive and–negative subjects remained significant, while the other 4 metabolites were now trends (p<0.09).

#### Sensitivity analysis with meta-data

Many of the subjects were receiving medications that might affect metabolic profiles. As expected, the PTSD group took more medication than did the control group across all medications (Table 1). In the Discovery Group, 40.4% of the PTSD group and 17.6% of the control group took some type of medication (chi-square, p <0.01), and in the Test Group, 32.2% of the PTSD group and 16.1% of the control group sook some type of medication (chi-square, p = 0.1).

Because the number of subjects taking each medication was too underpowered for ANCOVA analysis, medication effects were assessed in a sensitivity analysis in which t-tests were conducted to compare the PTSD-positive and -negative groups, with subjects taking particular medications excluded. When medications were grouped by type, the number of subjects regularly taking each was: anti-inflammatories (9), anti-depressants (17), statins (3), sedatives (7), anticonvulsants (3), antidiabetic agents (2), antibiotics (2) and beta-blockers (2) (Table 1). The reported metabolite differences between PTSD-positive and -negative subjects remained significant even in the subgroup of subjects not taking each type of medication (not shown). We also performed ANOVAs in both the Discovery and Test groups, excluding subjects taking *any* medication (Table 1). In the Discovery group, docosahexaenoate (DHA22:6n3) lost significance (p = 0.16), dimhomolinoleate 20:2n6, 10nonadecenoate 19:1n9, and eicosenoate 20:1 became trends (p<0.055), and the other metabolites remained significant. In the Test group, dihomolinoleate 20:2n6 (p = 0.19) and 10nonadecenoate 19:1n9 (p = 0.20) lost significance, while the other metabolites remained significant. In the Discovery group, we also compared metabolites from PTSD subjects taking *any* type of medication with PTSD subjects *not* taking *any* medication, and found that the PTSD positive and negative subjects do not show any significant difference (all p>0.3)

Among the PTSD subjects in the Discovery group, 27 were diagnosed with concurrent MDD, while no control subject was diagnosed with MDD. Nevertheless, using MDD status as a covariate did not affect the differences in metabolites seen between PTSD-positive and -negative subjects; furthermore, the reported metabolite differences between PTSD-positive and–negative subjects remained significant even in the subgroup of subjects without MDD. There were also no significant metabolite differences between PTSD subjects who did or did not also have MDD (all p > 0.3, not shown). These analyses suggest that MDD status did not play a significant role in explaining the between-group differences observed in PTSD-positive and PTSD-negative groups.

### Correlations with current CAPS scores

Using the current CAPS score as a continuous variable, none of the metabolites that were identified as significantly different between groups correlated significantly with the current CAPS score within the PTSD group.

## Discussion

In addition to traditional symptoms of PTSD (re-experiencing, avoidance, hyperarousal, negative thoughts or moods associated with the traumatic event [42]), individuals with PTSD also have a significantly increased medical burden, including higher rates of cardiovascular disease, metabolic syndrome, diabetes, autoimmune disease, and early morality [1, 41, 43, 44], suggesting that PTSD is both a behavioral and somatic disease. Certain processes have been proposed as contributing to the risk for these somatic diseases, including accelerated biological aging [45–56], sympathetic and glucocorticoid dysregulation [43, 57–61], metabolic changes [41, 45, 62, 63], inflammation [43, 57, 58, 64–71] and others. Some of these processes may involve changes in energy balance and mitochondrial function [72–77] that may be revealed by changes in metabolomic profiles.

**Fig 1:**
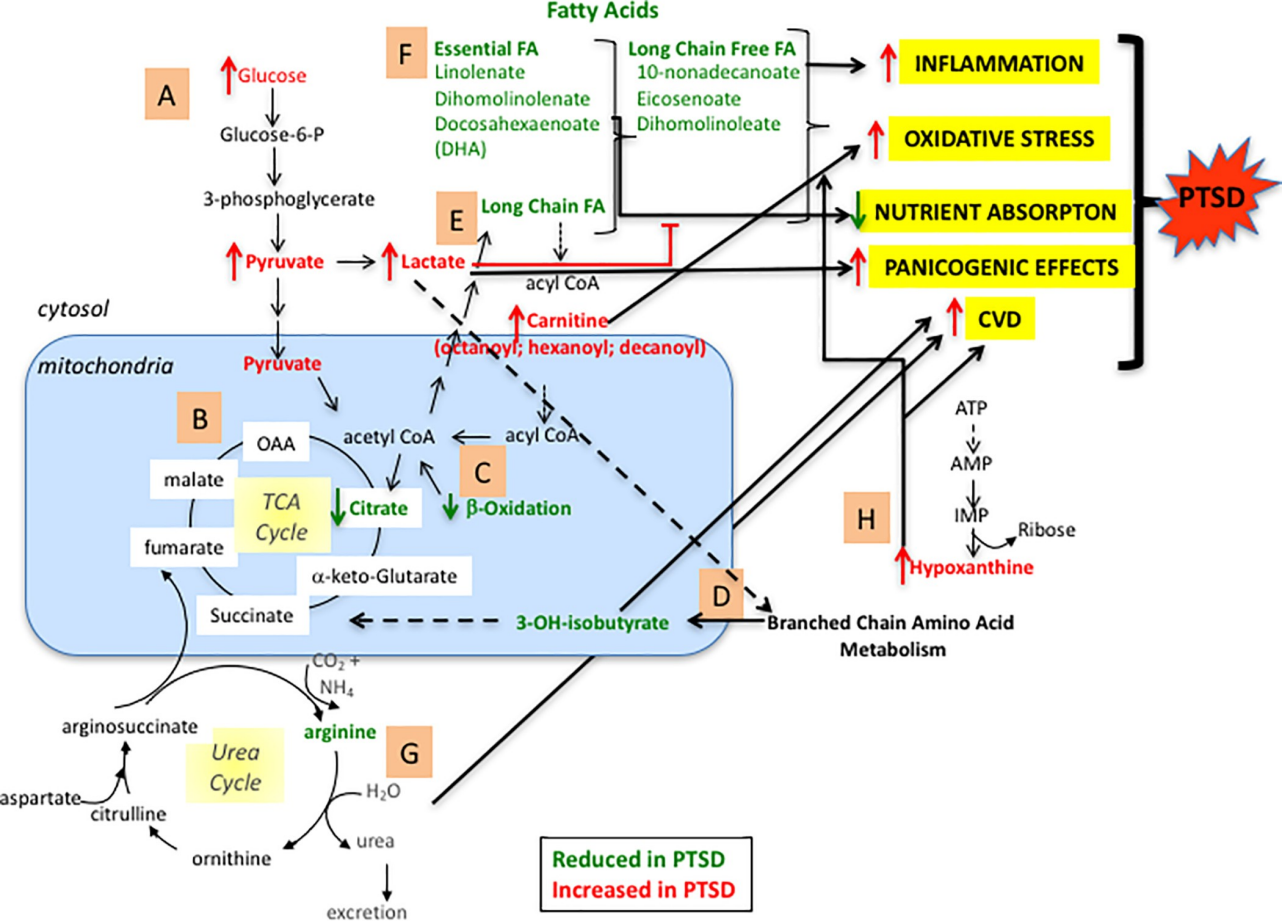
An overview of the metabolic imbalances in combat veterans with PTSD. The most significant metabolic alterations in PTSD can be organized into different biochemical pathways, including (**A**) glycolysis. (**B**) TCA cycle. (**C**) fatty acid oxidation, (**D**) branched chain amino acid pools, (**E**) lipid biosynthesis, (**F**) essential fatty acids, (**G**) urea cycle and (**H**) purine metabolisms. Metabolites that were ***elevated*** in PTSD in comparison to combat veterans who did not have PTSD are in ***red*** font, while metabolites that are ***reduced*** are in ***green*** font. Mitochondrial events are boxed in blue. Salient metabolic consequences that can potentially contribute to the manifestations of PTSD are highlighted in yellow, as discussed in the text.

Studies in mice [21] and humans [24–26] found dysregulation of genes affecting mitochondrial function in PTSD, hence we initially hypothesized that combat veterans with PTSD would have metabolite signatures indicating impaired mitochondrial function. In our Discovery group, we found significant differences between the metabolite profiles of male combat veterans who developed PTSD and those who did not, and these profiles were largely, but not uniformly, confirmed in our smaller Test group. The metabolite profiles are consistent with significant differences in mitochondrial function, energy utilization, and nutrient absorption or gut microbiota between these two groups of combat veterans. Alternatively, increases in inflammation and oxidative stress may lead to mitochondrial alteration or dysfunction [78]. In turn, these metabolite differences may contribute to increased inflammation, oxidative stress, anxiety, panic, obesity, metabolic syndrome, and cardiovascular disease, which are strongly associated with PTSD (Fig 1) [1, 19, 71, 79–81]. There are several mechanisms by which the altered metabolites, or the dysfunctional or compensatory pathways these metabolites reflect, may associate with PTSD.

### Lactate and pyruvate

The most robust metabolic between-group difference was the elevated concentrations of pyruvate and lactate, indicating enhanced anaerobic glycolysis in the PTSD-positive group. Decreased metabolites in the TCA cycle have been seen in brains of animal models of PTSD [20]. The pathophysiologcial significance of increased lactate in PTSD is unknown, but to the extent that lactate contributes to PTSD symptoms [82–92], several potential mechanisms are proposed, as described below.

GPR81, a cell-surface receptor coupled to G_i_ proteins, is activated by lactate and decreases intracellular cAMP [93–97]. GPR81 is highly expressed in adipocytes, suggesting a role in regulating lipolysis, and is also expressed in liver, kidney, skeletal muscle, spleen, and testis, where it may play a role in lipid metabolism or have other functions [97]. The elevated concentrations of lactate in PTSD may have synergistic anti-lipolytic effects, reducing the availability of long chain fatty acids for energy metabolism, which may also lead to the increased adiposity and BMI seen in these subjects. Elevated basal lactate concentrations are also associated with the development of insulin resistance [98, 99], which has been suggested to be due to decreased glucose utilization [100–102].

GPR81 is also found in neurons of the cerebral cortex, hippocampus (pyramidal and granule cells) and cerebellum (granule cells), where it can be activated by physiological concentrations (low mM) of lactate [103, 104]. In the cortex, GPR81 is found mainly on synaptic membranes of excitatory synapses, and is predominantly expressed on postsynaptic membranes. GPR81 is also enriched at the blood-brain barrier, further suggesting that lactate may play a role in signaling in the brain. Lactate, via GPR81, may act as a volume transmitter, linking neuronal activity, cerebral energy metabolism, and energy substrate availability [103, 104]. This occurs through regulating formation of cAMP and by adjusting the NADH/NAD ratio. Thus, lactate is a mediator of metabolic information in addition to being a metabolic substrate [105].

While the molecular mechanism is unknown, lactate can predispose to panic attacks in susceptible subjects [82–91], and patients with a panic disorder may have elevated brain lactate responses to metabolic challenges [92]. Lactate exerts excitatory effects on neuronal activity [106, 107]; thus, increased hippocampal firing, a direct action of lactate on the CNS, may contribute to lactate-induced panic.

Lactate also stimulates neuronal expression of genes related to synaptic plasticity (e.g. *Arc*, *c-Fos*, and *Zif268*), via NMDA receptors and their downstream signaling cascade to Erk1/2 in mouse primary neuronal cultures and *in vivo* [108]. Lactate can potentiate currents mediated by NMDA receptors *via* increased intracellular calcium or increased intraneuronal concentrations of NADH. By contrast, lactate binding to GPR81 may also directly inhibit both glutamatergic and GABA-ergic neuronal function by inhibiting the frequency of calcium transients, thus reducing the frequency of neuronal firing [109]. Elevated lactate and pyruvate decrease the cellular NADH/NAD ratio, which in turn regulates various *clock* genes [110], which may contribute to the disordered sleep [111] and glucocorticoid sensitivity [112] associated with PTSD. Our peripheral measurements of plasma lactate, however, may not completely reflect intracerebral lactate concentrations.

### Fatty acids

Fatty acids can be both pro- and anti-inflammatory [113]. Saturated free fatty acids are involved in the inflammatory response *via* toll-like receptors [114]. Because we found no differences in plasma concentrations of saturated fatty acids in PTSD versus controls, it is unlikely that the increased inflammation, previously reported in our PTSD subjects [69, 70] is due to increased saturated fatty acids stimulating toll-like receptors. However, there was a significant *decrease* in several *unsaturated* fatty acids in PTSD subjects. Dietary long chain polyunsaturated fatty acids are ligands of the nuclear peroxisome proliferator-activated receptors (PPAR). Binding to PPAR-α, -γ, and -δ suppresses expression of sterol regulatory element-binding proteins, nuclear transcription factor NFκB, and other transcription factors that regulate expression of genes involved in intermediary metabolism, thermoregulation, energy partitioning, growth, differentiation, and inflammatory responses [115–119]. Hence, reduced concentrations of unsaturated fatty acids could lead to enhanced inflammation and a variety of other effects in PTSD.

In addition to fatty acids being ligands for PPAR receptors, the omega-3 fatty acids are ligands for the G-protein coupled receptor GPR120 [120, 121], and are protective and anti-inflammatory [120, 121]. In obese mice, stimulation of GPR120 by omega-3 fatty acids inhibits inflammatory signaling and improves insulin sensitivity [121, 122]. We found low concentrations of the omega-3 unsaturated fatty acid docosahexaenoic acid (DHA) in PTSD subjects, raising the possibility that its reduced plasma concentration may contribute to inflammation in these PTSD subjects [69, 70]. Although not assessed directly, reduced concentrations of DHA may also contribute to reduced insulin sensitivity seen in our subjects [41].

Studies in humans [123–127] and rodents [128–134] indicate that omega-3 fatty acid deficiency may be associated with a variety of neuropsychiatric illnesses, including attention deficit hyperactivity disorder, depression, schizophrenia, autism spectrum disorders, and anxiety. Our data are consistent with animal studies showing that chronic social defeat stress [135] and variable, intermittent social defeat stress [136], models of human PTSD, disrupts regulation of lipid synthesis, including reduced levels of non-esterified fatty acids, increased levels of cholesterol and LDL cholesterol, and reduced fatty acid oxidation. Reduced abundance of omega-3 fatty acids in the CNS may reduce neurotransmission, especially by the dopaminergic and serotonergic systems, by affecting membrane fluidity and related receptor functions, thereby ultimately affecting brain structure and function [131, 137]. Based on promising pilot results [138], a clinical trial of omega-3 fatty acids has been started in patients with PTSD [139].

### Hypoxanthine

Hypoxanthine, a naturally occurring purine derivative that is involved in the salvage pathway for purine synthesis, has been shown to stimulate oxidative stress [140, 141], and elevated concentrations have been implicated in fear in dogs [142]. Hypoxanthine is also high in fecal samples in a chronic variable stress rat model of depression [143]. Smoking [144] and heavy drinking [145] are also associated with elevated levels of hypoxanthine. In our subjects, however, the increases in hypoxanthine in the PTSD group were not accounted for by smoking, as indicated by cotinine levels. In apolipoprotein E-deficient mice and cells, hypoxanthine also induces cholesterol accumulation and stimulates atherosclerosis through alterations in lipid transport enzymes, independent of conversion to xanthine and uric acid [146], a known risk for cardiovascular disease. Indeed, elevated hypoxanthine levels have been reported in human myocardial infarction [147], a condition with higher prevalence in individuals with PTSD.

### Limitations and strengths

There are three significant limitations to our study. First, the study utilized modest sample sizes, although this is the largest human PTSD metabolomics study published to date. Second, this study utilized only male combat trauma-exposed subjects. Thus, our findings should not be extrapolated uncritically to females with PTSD or to individuals of either sex with non-combat-related PTSD. Third, since this was a cross-sectional study, based on single time-point for blood and behavioral measurements, we cannot assess any causal relationship or variability in the measures over time. Being an exploratory study, we did not correct for multiple hypothesis testing. However, the q values (which assess the significance of the false discovery rate), the identification of different metabolites that exist within specific pathways, and the replication of our strongest results with a second group of subjects suggest that the metabolites identified are indeed significantly different between groups. Finally, it is possible that other genetic and epigenetic risks may contribute to the metabolomic differences between PTSD positive and negative subjects.

Among the strengths of the study, first, we used well-characterized, young, combat-exposed veterans and excluded subjects with significant traumatic brain injury or current un-controlled medical illness. Due to the deep phenotyping of our subjects, we were able to account for metabolomics effects secondary to several health (e.g. fasting blood sugar, HbA1c, tobacco use) medication, comorbidity, and anthropometric (e.g. BMI and waist-to-hip ratio) issues. Although many of our subjects were receiving various medications or had concomitant controlled medical illnesses, sensitivity analyses showed that medication and concomitant medical illnesses had little effect on the metabolic profiles between groups of subjects, and analysis of only subjects who were taking no medications gave similar results. Many of our PTSD subjects also had MDD; however, covarying for MDD did not change the results, and more importantly, the metabolite differences remained significant even when comparing only subjects without comorbid MDD. Nonetheless, we cannot rule out the possibility that comorbid MDD influenced some of the observed metabolic differences. Second, we used combat trauma-exposed veterans who did not develop PTSD as our control group, thus eliminating the contribution of prior combat trauma exposure *per se* to our findings. However, use of this sample as a control group may have resulted in selecting a particularly resilient control sample. Total and subcategory scores of early trauma (ETI) showed no differences between our PTSD positive and negative subjects, suggesting that the metabolomic differences we identified between groups was not due to early life trauma prior to combat. However, we did not have an assessment of prior adulthood traumas, which may have contributed to a different lifetime “trauma load” between groups. Third, and most importantly, we confirmed and validated the initial findings of our Discovery group in a wholly separate Test group, greatly limiting the Type I errors that frequently compromise metabolomics studies [148, 149].

### Conclusions

We have identified several metabolites and metabolic pathways that may distinguish male combat-exposed PTSD-positive and -negative subjects. These metabolites and metabolic pathways were different from those seen in a study of male and female civilian PTSD [18], which identified seven phospholipids (four of which were phosphatidyl ethanolamines and were elevated in PTSD) two fatty acid metabolites (reduced in PTSD, and different from those identified in the current study), two nucleosides (reduced in PTSD), three bile acids and derivatives (two reduced, one increased in PTSD), one monosaccharide (reduced in PTSD) and one antioxidant (reduced in PTSD) [18], suggesting that sex and the type of trauma, and perhaps comorbid medical illnesses (other than autoimmune disease and infection) and medications, may influence metabolic features. Alternatively, the metabolomic methodologies used by these two studies differed, and may not have had overlapping analyte identification, rather than necessarily having found different metabolites. Even though the Discovery study was designed to be exploratory and hypothesis-generating, many of the strongest findings were replicated in our smaller Test group. Nevertheless, our results must be replicated in other studies with larger and more diverse samples. Similar studies in female combat trauma-exposed veterans are critically needed, as sex may moderate metabolic function [150]. PTSD appears to share some metabolomic features with cardiovascular disease [151], Alzheimer’s disease [7, 8, 152], diabetes [17], multiple sclerosis [153], and depression [2, 4]. However, the overall metabolomic profiles of those diseases differ from those in our study, so it remains to be determined whether the metabolomic changes identified in our subjects are unique or specific to PTSD. In accordance with recent Research Domain Criteria (RDoC) for neuropsychiatric illnesses [154], it is possible that the metabolomic abnormalities identified here are trans-diagnostic and may map onto specific symptoms or disease characteristics more so than to specific DSM diagnoses [155]. Therapies targeting some of these apparently dysregulated metabolic pathways or perhaps targeting mitochondrial function [71, 156] may provide treatment for some pathologic aspects of PTSD, both behavioral and somatic. The fact that the abnormalities we observed were seen in young, somatically healthy individuals with PTSD raises the possibility that they precede and presage later somatic illness, suggesting the possibility of early identification and prophylactic treatment. Indeed, it is unknown whether these abnormalities are sequellae of PTSD or, rather, are pre-existing risk factors for developing PTSD. Therapies targeting mitochondrial dysfunction have been used in animals and humans with Parkinson’s, Huntington’s and Alzheimer’s diseases [157–160]. Conversely, effective PTSD treatments such as SSRI antidepressants [161] may have beneficial effects on these dysregulated biochemical pathways, in addition to the observed behavioral manifestations of PTSD, as noted in an animal study where fluoxetine pre-treatment averted some physiological sequellae of stress (energy metabolism) [20]. Our findings raise the possibility that metabolomic differences, or the processes they reflect, underlie some of the somatic illnesses seen more commonly in PTSD, and that they may contribute to biomarker-based personalized ways of tracking and treating underlying pathophysiology in PTSD.

## Supporting information

S1 File Supplementary methods and results metabolomics in PTSD. Table A. Analytes identified in the discovery group that were not significantly different between PTSD positive and PTSD negative subjects Figure A. Barplot of metabolite validation success fraction over 1000 permutations of discovery and test groups.
(DOCX)

S1 Dataset Metabolomics PTSD database PLoS One. This file contains the primary demographic and metabolomic data reported in this manuscript.
(XLSX)

## References

[1] Levine AB, Levine LM, Levine TB. Posttraumatic stress disorder and cardiometabolic disease. Cardiology. 2014;127(1):1–19. Epub 2013/10/26. 10.1159/000354910 . 24157651

[2] Quinones MP, Kaddurah-Daouk R. Metabolomics tools for identifying biomarkers for neuropsychiatric diseases. Neurobiology of disease. 2009;35(2):165–76. Epub 2009/03/24. 10.1016/j.nbd.2009.02.019 . 19303440

[3] Rozen S, Cudkowicz ME, Bogdanov M, Matson WR, Kristal BS, Beecher C, et al Metabolomic analysis and signatures in motor neuron disease. Metabolomics: Official journal of the Metabolomic Society. 2005;1(2):101–8. Epub 2008/09/30. 10.1007/s11306-005-4810-1 18820733 PMC2553219

[4] Paige LA, Mitchell MW, Krishnan KR, Kaddurah-Daouk R, Steffens DC. A preliminary metabolomic analysis of older adults with and without depression. International journal of geriatric psychiatry. 2007;22(5):418–23. Epub 2006/10/19. 10.1002/gps.1690 . 17048218

[5] Holmes E, Tsang TM, Huang JT, Leweke FM, Koethe D, Gerth CW, et al Metabolic profiling of CSF: evidence that early intervention may impact on disease progression and outcome in schizophrenia. PLoS medicine. 2006;3(8):e327 Epub 2006/08/29. 10.1371/journal.pmed.0030327 16933966 PMC1551919

[6] Kaddurah-Daouk R, McEvoy J, Baillie RA, Lee D, Yao JK, Doraiswamy PM, et al Metabolomic mapping of atypical antipsychotic effects in schizophrenia. Molecular psychiatry. 2007;12(10):934–45. Epub 2007/04/19. 10.1038/sj.mp.4002000 . 17440431

[7] Han X, Rozen S, Boyle SH, Hellegers C, Cheng H, Burke JR, et al Metabolomics in early Alzheimer's disease: identification of altered plasma sphingolipidome using shotgun lipidomics. PloS one. 2011;6(7):e21643 Epub 2011/07/23. 10.1371/journal.pone.0021643 21779331 PMC3136924

[8] Kaddurah-Daouk R, Rozen S, Matson W, Han X, Hulette CM, Burke JR, et al Metabolomic changes in autopsy-confirmed Alzheimer's disease. Alzheimer's & dementia: the journal of the Alzheimer's Association. 2011;7(3):309–17. Epub 2010/11/16. 10.1016/j.jalz.2010.06.001 21075060 PMC3061205

[9] Bogdanov M, Matson WR, Wang L, Matson T, Saunders-Pullman R, Bressman SS, et al Metabolomic profiling to develop blood biomarkers for Parkinson's disease. Brain: a journal of neurology. 2008;131(Pt 2):389–96. Epub 2008/01/29. 10.1093/brain/awm304 . 18222993

[10] Underwood BR, Broadhurst D, Dunn WB, Ellis DI, Michell AW, Vacher C, et al Huntington disease patients and transgenic mice have similar pro-catabolic serum metabolite profiles. Brain: a journal of neurology. 2006;129(Pt 4):877–86. Epub 2006/02/09. 10.1093/brain/awl027 . 16464959

[11] Koenig AM, Karabatsiakis A, Stoll T, Wilker S, Hennessy T, Hill MM, et al Serum profile changes in postpartum women with a history of childhood maltreatment: a combined metabolite and lipid fingerprinting study. Sci Rep. 2018;8(1):3468 10.1038/s41598-018-21763-6 29472571 PMC5823924

[12] Hadrevi J, Jonsdottir IH, Jansson PA, Eriksson JW, Sjors A. Plasma metabolomic patterns in patients with exhaustion disorder. Stress. 2018:1–10. 10.1080/10253890.2018.1494150 . 30084722

[13] Brindle JT, Antti H, Holmes E, Tranter G, Nicholson JK, Bethell HW, et al Rapid and noninvasive diagnosis of the presence and severity of coronary heart disease using 1H-NMR-based metabonomics. Nature medicine. 2002;8(12):1439–44. Epub 2002/11/26. 10.1038/nm802 . 12447357

[14] Sabatine MS, Liu E, Morrow DA, Heller E, McCarroll R, Wiegand R, et al Metabolomic identification of novel biomarkers of myocardial ischemia. Circulation. 2005;112(25):3868–75. Epub 2005/12/14. 10.1161/CIRCULATIONAHA.105.569137 . 16344383

[15] Lu J, Xie G, Jia W, Jia W. Metabolomics in human type 2 diabetes research. Frontiers of medicine. 2013;7(1):4–13. Epub 2013/02/05. 10.1007/s11684-013-0248-4 . 23377891

[16] Milburn MV, Lawton KA. Application of metabolomics to diagnosis of insulin resistance. Annual review of medicine. 2013;64:291–305. Epub 2013/01/19. 10.1146/annurev-med-061511-134747 . 23327524

[17] Wang TJ, Larson MG, Vasan RS, Cheng S, Rhee EP, McCabe E, et al Metabolite profiles and the risk of developing diabetes. Nature medicine. 2011;17(4):448–53. Epub 2011/03/23. 10.1038/nm.2307 21423183 PMC3126616

[18] Karabatsiakis A, Hamuni G, Wilker S, Kolassa S, Renu D, Kadereit S, et al Metabolite profiling in posttraumatic stress disorder. J Mol Psychiatry. 2015;3(1):2 10.1186/s40303-015-0007-3 25848535 PMC4367823

[19] Frayne SM, Chiu VY, Iqbal S, Berg EA, Laungani KJ, Cronkite RC, et al Medical care needs of returning veterans with PTSD: their other burden. J Gen Intern Med. 2011;26(1):33–9. 10.1007/s11606-010-1497-4 20853066 PMC3024098

[20] Kao CY, He Z, Henes K, Asara JM, Webhofer C, Filiou MD, et al Fluoxetine treatment rescues energy metabolism pathway alterations in a posttraumatic stress disorder mouse model. Mol Neuropsychiatry. 2016;2(1):46–59. 10.1159/000445377 27606320 PMC4996011

[21] Li XM, Han F, Liu DJ, Shi YX. Single-prolonged stress induced mitochondrial-dependent apoptosis in hippocampus in the rat model of post-traumatic stress disorder. Journal of chemical neuroanatomy. 2010;40(3):248–55. Epub 2010/07/14. 10.1016/j.jchemneu.2010.07.001 . 20624456

[22] Liu D, Xiao B, Han F, Wang E, Shi Y. Single-prolonged stress induces apoptosis in dorsal raphe nucleus in the rat model of posttraumatic stress disorder. BMC psychiatry. 2012;12:211 Epub 2012/11/28. 10.1186/1471-244X-12-211 23181934 PMC3549289

[23] Li H, Li X, Smerin SE, Zhang L, Jia M, Xing G, et al Mitochondrial Gene Expression Profiles and Metabolic Pathways in the Amygdala Associated with Exaggerated Fear in an Animal Model of PTSD. Front Neurol. 2014;5:164 10.3389/fneur.2014.00164 25295026 PMC4172054

[24] Hammamieh R, Chakraborty N, Gautam A, Muhie S, Yang R, Donohue D, et al Whole-genome DNA methylation status associated with clinical PTSD measures of OIF/OEF veterans. Transl Psychiatry. 2017;7(7):e1169 10.1038/tp.2017.129 28696412 PMC5538114

[25] Su YA, Wu J, Zhang L, Zhang Q, Su DM, He P, et al Dysregulated mitochondrial genes and networks with drug targets in postmortem brain of patients with posttraumatic stress disorder (PTSD) revealed by human mitochondria-focused cDNA microarrays. International journal of biological sciences. 2008;4(4):223–35. Epub 2008/08/12. 18690294 10.7150/ijbs.4.223 PMC2500154

[26] Zhang L, Hu XZ, Benedek DM, Fullerton CS, Forsten RD, Naifeh JA, et al Dysregulated mitochondria-focused genes in US military service members with PTSD. European Journal of Psychotraumatology. 2012;3 10.3402/ejpt.v3i0.19309 WOS:000208868500178.

[27] Boeck C, Koenig AM, Schury K, Geiger ML, Karabatsiakis A, Wilker S, et al Inflammation in adult women with a history of child maltreatment: The involvement of mitochondrial alterations and oxidative stress. Mitochondrion. 2016;30:197–207. 10.1016/j.mito.2016.08.006 . 27530300

[28] Karabatsiakis A, Böck C, Salinas-Manrique J, Kolassa S, Calzia E, Dietrich DE, et al Mitochondrial respiration in peripheral blood mononuclear cells correlates with depressive subsymptoms and severity of major depression. Transl Psychiatry. 2014;4:e397. 10.1038/tp.2014.44 PMC4080325 26126180

[29] Thakur GS, Daigle BJ Jr., Dean KR, Zhang Y, Rodriguez-Fernandez M, Hammamieh R, et al Systems biology approach to understanding post-traumatic stress disorder. Mol Biosyst. 2015;11(4):980–93. 10.1039/c4mb00404c . 25627823

[30] Blake DD, Weathers FW, Nagy LM, Kaloupek DG, Gusman FD, Charney DS, et al The development of a Clinician-Administered PTSD Scale. Journal of traumatic stress. 1995;8(1):75–90. Epub 1995/01/01. . 7712061 10.1007/BF02105408

[31] Corrigan JD, Bogner J. Initial reliability and validity of the Ohio State University TBI Identification Method. The Journal of head trauma rehabilitation. 2007;22(6):318–29. Epub 2007/11/21. 10.1097/01.HTR.0000300227.67748.77 . 18025964

[32] First MB. Structured clinical interview for DSM-IV axis I disorders SCID-I: clinician version, administration booklet. Washington, DC: American Psychiatric Press; 1997.

[33] Beck AT, Steer RA, Brown GK. Manual for Beck Depression Inventory-II. San Antonio, TX: Psychological Corporation; 1996.

[34] Dehaven CD, Evans AM, Dai H, Lawton KA. Organization of GC/MS and LC/MS metabolomics data into chemical libraries. Journal of cheminformatics. 2010;2(1):9 Epub 2010/10/20. 10.1186/1758-2946-2-9 20955607 PMC2984397

[35] Evans AM, DeHaven CD, Barrett T, Mitchell M, Milgram E. Integrated, nontargeted ultrahigh performance liquid chromatography/electrospray ionization tandem mass spectrometry platform for the identification and relative quantification of the small-molecule complement of biological systems. Analytical chemistry. 2009;81(16):6656–67. Epub 2009/07/25. 10.1021/ac901536h . 19624122

[36] Ohta T, Masutomi N, Tsutsui N, Sakairi T, Mitchell M, Milburn MV, et al Untargeted metabolomic profiling as an evaluative tool of fenofibrate-induced toxicology in Fischer 344 male rats. Toxicologic pathology. 2009;37(4):521–35. Epub 2009/05/22. 10.1177/0192623309336152 . 19458390

[37] Blom G. Statistical estimates and transformed beta-variables. NY, NY: J. Wiley and Sons; 1958.

[38] Storey JD, Tibshirani R. Statistical significance for genomewide studies. Proceedings of the National Academy of Sciences of the United States of America. 2003;100(16):9440–5. Epub 2003/07/29. 10.1073/pnas.1530509100 12883005 PMC170937

[39] Hamer M, O'Donovan G, Stensel D, Stamatakis E. Normal-Weight Central Obesity and Risk for Mortality. Ann Intern Med. 2017;166(12):917–8. 10.7326/L17-0022 . 28437799

[40] Agarwal A, Banerjee A, Banerjee UC. Xanthine oxidoreductase: a journey from purine metabolism to cardiovascular excitation-contraction coupling. Crit Rev Biotechnol. 2011;31(3):264–80. 10.3109/07388551.2010.527823 . 21774633

[41] Blessing E, Reus VI, Mellon SH, Wolkowitz OM, Flory JD, Bierer LM, et al Biological predictors of insulin resistance associated with posttraumatic stress disorder in young military veterans. Psychoneuroendocrinology. 2017;82:91–7. 10.1016/j.psyneuen.2017.04.016 . 28521179

[42] Association AP, editor. Diagnostic and statistical manual of mental disorders. 5th ed Arlington, VA: American Psychiatric Publishing; 2013.

[43] O'Donovan A, Cohen BE, Seal KH, Bertenthal D, Margaretten M, Nishimi K, et al Elevated risk for autoimmune disorders in iraq and afghanistan veterans with posttraumatic stress disorder. Biological psychiatry. 2015;77(4):365–74. 10.1016/j.biopsych.2014.06.015 25104173 PMC4277929

[44] Pacella ML, Hruska B, Delahanty DL. The physical health consequences of PTSD and PTSD symptoms: a meta-analytic review. J Anxiety Disord. 2013;27(1):33–46. 10.1016/j.janxdis.2012.08.004 . 23247200

[45] Wolf EJ, Schnurr PP. PTSD-Related Cardiovascular Disease and Accelerated Cellular Aging. Psychiatr Ann. 2016;46:527–32. 27990033 10.3928/00485713-20160729-01 PMC5154362

[46] Wolf EJ, Logue MW, Morrison FG, Wilcox ES, Stone A, Schichman SA, et al Posttraumatic psychopathology and the pace of the epigenetic clock: a longitudinal investigation. Psychol Med. 2018:1–10. 10.1017/S0033291718001411 . 29897034 PMC6292741

[47] Mehta D, Bruenig D, Lawford B, Harvey W, Carrillo-Roa T, Morris CP, et al Accelerated DNA methylation aging and increased resilience in veterans: The biological cost for soldiering on. Neurobiol Stress. 2018;8:112–9. 10.1016/j.ynstr.2018.04.001 29888306 PMC5991315

[48] Kuffer AL, O'Donovan A, Burri A, Maercker A. Posttraumatic Stress Disorder, Adverse Childhood Events, and Buccal Cell Telomere Length in Elderly Swiss Former Indentured Child Laborers. Front Psychiatry. 2016;7:147 10.3389/fpsyt.2016.00147 27630582 PMC5005955

[49] Moreno-Villanueva M, Morath J, Vanhooren V, Elbert T, Kolassa S, Libert C, et al N-glycosylation profiling of plasma provides evidence for accelerated physiological aging in post-traumatic stress disorder. Transl Psychiatry. 2013;3:e320 10.1038/tp.2013.93 24169639 PMC3818009

[50] Roberts AL, Koenen KC, Chen Q, Gilsanz P, Mason SM, Prescott J, et al Posttraumatic stress disorder and accelerated aging: PTSD and leukocyte telomere length in a sample of civilian women. Depression and anxiety. 2017;34(5):391–400. 10.1002/da.22620 28380289 PMC5848097

[51] Stein JY, Levin Y, Uziel O, Abumock H, Solomon Z. Traumatic stress and cellular senescence: The role of war-captivity and homecoming stressors in later life telomere length. J Affect Disord. 2018;238:129–35. 10.1016/j.jad.2018.05.037 . 29879607

[52] Wolf EJ, Maniates H, Nugent N, Maihofer AX, Armstrong D, Ratanatharathorn A, et al Traumatic stress and accelerated DNA methylation age: A meta-analysis. Psychoneuroendocrinology. 2018;92:123–34. 10.1016/j.psyneuen.2017.12.007 29452766 PMC5924645

[53] Wolf EJ, Morrison FG. Traumatic Stress and Accelerated Cellular Aging: From Epigenetics to Cardiometabolic Disease. Curr Psychiatry Rep. 2017;19(10):75 10.1007/s11920-017-0823-5 28852965 PMC5588711

[54] Zhang L, Hu XZ, Li X, Li H, Smerin S, Russell D, et al Telomere length—a cellular aging marker for depression and Post-traumatic Stress Disorder. Medical hypotheses. 2014;83(2):182–5. 10.1016/j.mehy.2014.04.033 . 24875221

[55] Konjevod M, Tudor L, Svob Strac D, Nedic Erjavec G, Barbas C, Zarkovic N, et al Metabolomic and glycomic findings in posttraumatic stress disorder. Prog Neuropsychopharmacol Biol Psychiatry. 2019;88:181–93. 10.1016/j.pnpbp.2018.07.014 . 30025792

[56] Verhoeven JE, Yang R, Wolkowitz OM, Bersani FS, Lindqvist D, Mellon SH, et al Epigenetic Age in Male Combat-Exposed War Veterans: Associations with Posttraumatic Stress Disorder Status. Molecular Neuropsychiatry. 2018;4:102–11. 10.1159/000491431 PMC6206951 30397597

[57] Gill J, Luckenbaugh D, Charney D, Vythilingam M. Sustained elevation of serum interleukin-6 and relative insensitivity to hydrocortisone differentiates posttraumatic stress disorder with and without depression. Biological psychiatry. 2010;68(11):999–1006. 10.1016/j.biopsych.2010.07.033 . 20951370

[58] Pace TW, Heim CM. A short review on the psychoneuroimmunology of posttraumatic stress disorder: from risk factors to medical comorbidities. Brain, behavior, and immunity. 2011;25(1):6–13. 10.1016/j.bbi.2010.10.003 . 20934505

[59] Wheler GH, Brandon D, Clemons A, Riley C, Kendall J, Loriaux DL, et al Cortisol production rate in posttraumatic stress disorder. The Journal of clinical endocrinology and metabolism. 2006;91(9):3486–9. Epub 2006/06/22. 10.1210/jc.2006-0061 . 16787989

[60] Yehuda R, Bierer LM, Andrew R, Schmeidler J, Seckl JR. Enduring effects of severe developmental adversity, including nutritional deprivation, on cortisol metabolism in aging Holocaust survivors. Journal of psychiatric research. 2009;43(9):877–83. Epub 2009/01/24. 10.1016/j.jpsychires.2008.12.003 19162277 PMC2749458

[61] Yehuda R, Bierer LM, Sarapas C, Makotkine I, Andrew R, Seckl JR. Cortisol metabolic predictors of response to psychotherapy for symptoms of PTSD in survivors of the World Trade Center attacks on September 11, 2001. Psychoneuroendocrinology. 2009;34(9):1304–13. Epub 2009/05/05. 10.1016/j.psyneuen.2009.03.018 19411143 PMC2785023

[62] Michopoulos V, Vester A, Neigh G. Posttraumatic stress disorder: A metabolic disorder in disguise? Exp Neurol. 2016;284(Pt B):220–9. 10.1016/j.expneurol.2016.05.038 27246996 PMC5056806

[63] Rosenbaum S, Stubbs B, Ward PB, Steel Z, Lederman O, Vancampfort D. The prevalence and risk of metabolic syndrome and its components among people with posttraumatic stress disorder: a systematic review and meta-analysis. Metabolism: clinical and experimental. 2015;64(8):926–33. 10.1016/j.metabol.2015.04.009 . 25982700

[64] Gola H, Engler H, Sommershof A, Adenauer H, Kolassa S, Schedlowski M, et al Posttraumatic stress disorder is associated with an enhanced spontaneous production of pro-inflammatory cytokines by peripheral blood mononuclear cells. BMC psychiatry. 2013;13:40 10.1186/1471-244X-13-40 23360282 PMC3574862

[65] Passos IC, Vasconcelos-Moreno MP, Costa LG, Kunz M, Brietzke E, Quevedo J, et al Inflammatory markers in post-traumatic stress disorder: a systematic review, meta-analysis, and meta-regression. Lancet Psychiatry. 2015;2(11):1002–12. 10.1016/S2215-0366(15)00309-0 . 26544749

[66] Wang Z, Mandel H, Levingston CA, Young MR. An exploratory approach demonstrating immune skewing and a loss of coordination among cytokines in plasma and saliva of Veterans with combat-related PTSD. Hum Immunol. 2016;77(8):652–7. 10.1016/j.humimm.2016.05.018 . 27216157 PMC5937020

[67] Wang Z, Young MR. PTSD, a Disorder with an Immunological Component. Front Immunol. 2016;7:219 10.3389/fimmu.2016.00219 27375619 PMC4893499

[68] Bersani FS, Wolkowitz OM, Lindqvist D, Yehuda R, Flory J, Bierer LM, et al Global arginine bioavailability, a marker of nitric oxide synthetic capacity, is decreased in PTSD and correlated with symptom severity and markers of inflammation. Brain, behavior, and immunity. 2016;52:153–60. 10.1016/j.bbi.2015.10.015 . 26515034

[69] Lindqvist D, Mellon SH, Dhabhar FS, Yehuda R, Marlene Grenon S, Flory JD, et al Increased pro-inflammatory milieu in combat related PTSD—a new cohort replication study. Brain, behavior, and immunity. 2016 10.1016/j.bbi.2016.09.012 . 27638184

[70] Lindqvist D, Wolkowitz OM, Mellon S, Yehuda R, Flory JD, Henn-Haase C, et al Proinflammatory milieu in combat-related PTSD is independent of depression and early life stress. Brain, behavior, and immunity. 2014;42:81–8. Epub 2014/06/15. 10.1016/j.bbi.2014.06.003 . 24929195

[71] Mellon SH, Gautam A, Hammamieh R, Jett M, Wolkowitz OM. Metabolism, Metabolomics, and Inflammation in Posttraumatic Stress Disorder. Biological psychiatry. 2018;83(10):866–75. 10.1016/j.biopsych.2018.02.007 . 29628193

[72] Zhang L, Zhou R, Li X, Ursano RJ, Li H. Stress-induced change of mitochondria membrane potential regulated by genomic and non-genomic GR signaling: a possible mechanism for hippocampus atrophy in PTSD. Medical hypotheses. 2006;66(6):1205–8. 10.1016/j.mehy.2005.11.041 . 16446049

[73] Xing G, Barry ES, Benford B, Grunberg NE, Li H, Watson WD, et al Impact of repeated stress on traumatic brain injury-induced mitochondrial electron transport chain expression and behavioral responses in rats. Front Neurol. 2013;4:196 10.3389/fneur.2013.00196 24376434 PMC3859919

[74] Naviaux RK. Metabolic features of the cell danger response. Mitochondrion. 2014;16:7–17. Epub 2013/08/29. 10.1016/j.mito.2013.08.006 . 23981537

[75] Amigo I, da Cunha FM, Forni MF, Garcia-Neto W, Kakimoto PA, Luevano-Martinez LA, et al Mitochondrial form, function and signalling in aging. Biochem J. 2016;473(20):3421–49. 10.1042/BCJ20160451 . 27729586

[76] Bunkar N, Bhargava A, Khare NK, Mishra PK. Mitochondrial anomalies: driver to age associated degenerative human ailments. Front Biosci (Landmark Ed). 2016;21:769–93. . 26709805 10.2741/4420

[77] Morava E, Kozicz T. Mitochondria and the economy of stress (mal)adaptation. Neurosci Biobehav Rev. 2013;37(4):668–80. 10.1016/j.neubiorev.2013.02.005 . 23415702

[78] Tschopp J. Mitochondria: Sovereign of inflammation? Eur J Immunol. 2011;41(5):1196–202. 10.1002/eji.201141436 . 21469137

[79] Wentworth BA, Stein MB, Redwine LS, Xue Y, Taub PR, Clopton P, et al Post-traumatic stress disorder: a fast track to premature cardiovascular disease? Cardiology in review. 2013;21(1):16–22. Epub 2012/06/22. 10.1097/CRD.0b013e318265343b . 22717656

[80] Maslov B, Marcinko D, Milicevic R, Babic D, Dordevic V, Jakovljevic M. Metabolic syndrome, anxiety, depression and suicidal tendencies in post-traumatic stress disorder and schizophrenic patients. Collegium antropologicum. 2009;33 Suppl 2:7–10. Epub 2010/02/03. . 20120396

[81] Player MS, Peterson LE. Anxiety disorders, hypertension, and cardiovascular risk: a review. International journal of psychiatry in medicine. 2011;41(4):365–77. Epub 2012/01/14. 10.2190/PM.41.4.f . 22238841

[82] Jensen CF, Keller TW, Peskind ER, McFall ME, Veith RC, Martin D, et al Behavioral and plasma cortisol responses to sodium lactate infusion in posttraumatic stress disorder. Annals of the New York Academy of Sciences. 1997;821:444–8. Epub 1997/06/21. . 9238226 10.1111/j.1749-6632.1997.tb48301.x

[83] Jensen CF, Keller TW, Peskind ER, McFall ME, Veith RC, Martin D, et al Behavioral and neuroendocrine responses to sodium lactate infusion in subjects with posttraumatic stress disorder. The American journal of psychiatry. 1997;154(2):266–8. Epub 1997/02/01. 10.1176/ajp.154.2.266 . 9016280

[84] Jensen CF, Peskind ER, Keller TW, McFall ME, Raskind MA. Comparison of sodium lactate-induced panic symptoms between panic disorder and posttraumatic stress disorder. Depression and anxiety. 1998;7(3):122–5. Epub 1998/07/10. . 9656092

[85] Knox D, Perrine SA, George SA, Galloway MP, Liberzon I. Single prolonged stress decreases glutamate, glutamine, and creatine concentrations in the rat medial prefrontal cortex. Neuroscience letters. 2010;480(1):16–20. Epub 2010/06/16. 10.1016/j.neulet.2010.05.052 20546834 PMC2902659

[86] Maddock RJ, Buonocore MH. MR Spectroscopic Studies of the Brain in Psychiatric Disorders. Current topics in behavioral neurosciences. 2012 Epub 2012/02/02. 10.1007/7854_2011_197 . 22294088

[87] Muhtz C, Wiedemann K, Kellner M. Panicogens in patients with Post-Traumatic Stress Disorder (PTSD). Current pharmaceutical design. 2012;18(35):5608–18. Epub 2012/05/29. . 22632476 10.2174/138161212803530817

[88] Perry D. 1st International Conference on Panic Attacks: diversity of theories and treatments. September 5–8, 2003, London. Expert opinion on pharmacotherapy. 2004;5(4):977–80. Epub 2004/04/23. 10.1517/14656566.5.4.977 . 15102580

[89] Rainey JM Jr., Aleem A, Ortiz A, Yeragani V, Pohl R, Berchou R. A laboratory procedure for the induction of flashbacks. The American journal of psychiatry. 1987;144(10):1317–9. Epub 1987/10/01. 10.1176/ajp.144.10.1317 . 3661765

[90] Stehberg J, Moraga-Amaro R, Salazar C, Becerra A, Echeverria C, Orellana JA, et al Release of gliotransmitters through astroglial connexin 43 hemichannels is necessary for fear memory consolidation in the basolateral amygdala. FASEB journal: official publication of the Federation of American Societies for Experimental Biology. 2012;26(9):3649–57. Epub 2012/06/06. 10.1096/fj.11-198416 . 22665389

[91] Strohle A, Holsboer F. Stress responsive neurohormones in depression and anxiety. Pharmacopsychiatry. 2003;36 Suppl 3:S207–14. Epub 2003/12/17. 10.1055/s-2003-45132 . 14677081

[92] Maddock RJ, Buonocore MH, Copeland LE, Richards AL. Elevated brain lactate responses to neural activation in panic disorder: a dynamic 1H-MRS study. Molecular psychiatry. 2009;14(5):537–45. Epub 2008/01/09. 10.1038/sj.mp.4002137 . 18180759

[93] Cai TQ, Ren N, Jin L, Cheng K, Kash S, Chen R, et al Role of GPR81 in lactate-mediated reduction of adipose lipolysis. Biochemical and biophysical research communications. 2008;377(3):987–91. Epub 2008/10/28. 10.1016/j.bbrc.2008.10.088 . 18952058

[94] Ahmed K, Tunaru S, Langhans CD, Hanson J, Michalski CW, Kolker S, et al Deorphanization of GPR109B as a receptor for the beta-oxidation intermediate 3-OH-octanoic acid and its role in the regulation of lipolysis. The Journal of biological chemistry. 2009;284(33):21928–33. Epub 2009/06/30. 10.1074/jbc.M109.019455 19561068 PMC2755917

[95] Ahmed K, Tunaru S, Offermanns S. GPR109A, GPR109B and GPR81, a family of hydroxy-carboxylic acid receptors. Trends in pharmacological sciences. 2009;30(11):557–62. Epub 2009/10/20. 10.1016/j.tips.2009.09.001 . 19837462

[96] Ahmed K, Tunaru S, Tang C, Muller M, Gille A, Sassmann A, et al An autocrine lactate loop mediates insulin-dependent inhibition of lipolysis through GPR81. Cell metabolism. 2010;11(4):311–9. Epub 2010/04/09. 10.1016/j.cmet.2010.02.012 . 20374963

[97] Liu C, Wu J, Zhu J, Kuei C, Yu J, Shelton J, et al Lactate inhibits lipolysis in fat cells through activation of an orphan G-protein-coupled receptor, GPR81. The Journal of biological chemistry. 2009;284(5):2811–22. Epub 2008/12/03. 10.1074/jbc.M806409200 . 19047060

[98] Lovejoy J, Newby FD, Gebhart SS, DiGirolamo M. Insulin resistance in obesity is associated with elevated basal lactate levels and diminished lactate appearance following intravenous glucose and insulin. Metabolism: clinical and experimental. 1992;41(1):22–7. Epub 1992/01/01. . 1538640 10.1016/0026-0495(92)90185-d

[99] Lovejoy J, Mellen B, Digirolamo M. Lactate generation following glucose ingestion: relation to obesity, carbohydrate tolerance and insulin sensitivity. International journal of obesity. 1990;14(10):843–55. Epub 1990/10/01. . 2269580

[100] Pearce FJ, Connett RJ. Effect of lactate and palmitate on substrate utilization of isolated rat soleus. The American journal of physiology. 1980;238(5):C149–59. Epub 1980/05/01. 10.1152/ajpcell.1980.238.5.C149 . 6990780

[101] Clark AS, Mitch WE, Goodman MN, Fagan JM, Goheer MA, Curnow RT. Dichloroacetate inhibits glycolysis and augments insulin-stimulated glycogen synthesis in rat muscle. The Journal of clinical investigation. 1987;79(2):588–94. Epub 1987/02/01. 10.1172/JCI112851 3543056 PMC424134

[102] Fisher AB, Dodia C. Lactate and regulation of lung glycolytic rate. The American journal of physiology. 1984;246(5 Pt 1):E426–9. Epub 1984/05/01. 10.1152/ajpendo.1984.246.5.E426 . 6720945

[103] Lauritzen F, Eid T, Bergersen LH. Monocarboxylate transporters in temporal lobe epilepsy: roles of lactate and ketogenic diet. Brain structure & function. 2013 Epub 2013/11/20. 10.1007/s00429-013-0672-x . 24248427

[104] Lauritzen KH, Morland C, Puchades M, Holm-Hansen S, Hagelin EM, Lauritzen F, et al Lactate Receptor Sites Link Neurotransmission, Neurovascular Coupling, and Brain Energy Metabolism. Cereb Cortex. 2013 Epub 2013/05/23. 10.1093/cercor/bht136 . 23696276

[105] Bergersen LH, Gjedde A. Is lactate a volume transmitter of metabolic states of the brain? Frontiers in neuroenergetics. 2012;4:5 Epub 2012/03/30. 10.3389/fnene.2012.00005 22457647 PMC3307048

[106] Bergold PJ, Pinkhasova V, Syed M, Kao HY, Jozwicka A, Zhao N, et al Production of panic-like symptoms by lactate is associated with increased neural firing and oxidation of brain redox in the rat hippocampus. Neuroscience letters. 2009;453(3):219–24. Epub 2009/05/12. 10.1016/j.neulet.2009.02.041 . 19429039

[107] von Pfostl V, Li J, Zaldivar D, Goense J, Zhang X, Serr N, et al Effects of lactate on the early visual cortex of non-human primates, investigated by pharmaco-MRI and neurochemical analysis. NeuroImage. 2012;61(1):98–105. Epub 2012/03/20. 10.1016/j.neuroimage.2012.02.082 . 22426350

[108] Yang J, Ruchti E, Petit JM, Jourdain P, Grenningloh G, Allaman I, et al Lactate promotes plasticity gene expression by potentiating NMDA signaling in neurons. Proceedings of the National Academy of Sciences of the United States of America. 2014;111(33):12228–33. Epub 2014/07/30. 10.1073/pnas.1322912111 25071212 PMC4143009

[109] Bozzo L, Puyal J, Chatton JY. Lactate modulates the activity of primary cortical neurons through a receptor-mediated pathway. PloS one. 2013;8(8):e71721 Epub 2013/08/21. 10.1371/journal.pone.0071721 23951229 PMC3741165

[110] Zhao X, Cho H, Yu RT, Atkins AR, Downes M, Evans RM. Nuclear receptors rock around the clock. EMBO Rep. 2014;15(5):518–28. 10.1002/embr.201338271 24737872 PMC4210094

[111] Koffel E, Khawaja IS, Germain A. Sleep Disturbances in Posttraumatic Stress Disorder: Updated Review and Implications for Treatment. Psychiatr Ann. 2016;46(3):173–6. 10.3928/00485713-20160125-01 27773950 PMC5068571

[112] Nader N, Chrousos GP, Kino T. Circadian rhythm transcription factor CLOCK regulates the transcriptional activity of the glucocorticoid receptor by acetylating its hinge region lysine cluster: potential physiological implications. FASEB journal: official publication of the Federation of American Societies for Experimental Biology. 2009;23(5):1572–83. 10.1096/fj.08-117697 19141540 PMC2669420

[113] Meijer K, de Vos P, Priebe MG. Butyrate and other short-chain fatty acids as modulators of immunity: what relevance for health? Current opinion in clinical nutrition and metabolic care. 2010;13(6):715–21. Epub 2010/09/09. 10.1097/MCO.0b013e32833eebe5 . 20823773

[114] Baker RG, Hayden MS, Ghosh S. NF-kappaB, inflammation, and metabolic disease. Cell metabolism. 2011;13(1):11–22. Epub 2011/01/05. 10.1016/j.cmet.2010.12.008 21195345 PMC3040418

[115] Ahmadian M, Suh JM, Hah N, Liddle C, Atkins AR, Downes M, et al PPARgamma signaling and metabolism: the good, the bad and the future. Nature medicine. 2013;19(5):557–66. Epub 2013/05/09. 10.1038/nm.3159 23652116 PMC3870016

[116] Duplus E, Forest C. Is there a single mechanism for fatty acid regulation of gene transcription? Biochemical pharmacology. 2002;64(5–6):893–901. Epub 2002/09/06. . 12213584 10.1016/s0006-2952(02)01157-7

[117] Clarke SD. The multi-dimensional regulation of gene expression by fatty acids: polyunsaturated fats as nutrient sensors. Current opinion in lipidology. 2004;15(1):13–8. Epub 2004/05/29. . 15166803 10.1097/00041433-200402000-00004

[118] Jump DB. Fatty acid regulation of gene transcription. Critical reviews in clinical laboratory sciences. 2004;41(1):41–78. Epub 2004/04/14. 10.1080/10408360490278341 . 15077723

[119] Lapillonne A, Clarke SD, Heird WC. Polyunsaturated fatty acids and gene expression. Current opinion in clinical nutrition and metabolic care. 2004;7(2):151–6. Epub 2004/04/13. . 15075705 10.1097/00075197-200403000-00008

[120] Hirasawa A, Tsumaya K, Awaji T, Katsuma S, Adachi T, Yamada M, et al Free fatty acids regulate gut incretin glucagon-like peptide-1 secretion through GPR120. Nature medicine. 2005;11(1):90–4. Epub 2004/12/28. 10.1038/nm1168 . 15619630

[121] Oh DY, Talukdar S, Bae EJ, Imamura T, Morinaga H, Fan W, et al GPR120 is an omega-3 fatty acid receptor mediating potent anti-inflammatory and insulin-sensitizing effects. Cell. 2010;142(5):687–98. Epub 2010/09/04. 10.1016/j.cell.2010.07.041 20813258 PMC2956412

[122] Oh da Y, Walenta E, Akiyama TE, Lagakos WS, Lackey D, Pessentheiner AR, et al A Gpr120-selective agonist improves insulin resistance and chronic inflammation in obese mice. Nature medicine. 2014;20(8):942–7. Epub 2014/07/07. 10.1038/nm.3614 24997608 PMC4126875

[123] Janssen CI, Kiliaan AJ. Long-chain polyunsaturated fatty acids (LCPUFA) from genesis to senescence: the influence of LCPUFA on neural development, aging, and neurodegeneration. Progress in lipid research. 2014;53:1–17. Epub 2013/12/18. 10.1016/j.plipres.2013.10.002 . 24334113

[124] McNamara RK, Strawn JR. Role of Long-Chain Omega-3 Fatty Acids in Psychiatric Practice. PharmaNutrition. 2013;1(2):41–9. Epub 2013/04/23. 10.1016/j.phanu.2012.10.004 23607087 PMC3628733

[125] Milte CM, Parletta N, Buckley JD, Coates AM, Young RM, Howe PR. Increased Erythrocyte Eicosapentaenoic Acid and Docosahexaenoic Acid Are Associated With Improved Attention and Behavior in Children With ADHD in a Randomized Controlled Three-Way Crossover Trial. Journal of attention disorders. 2013 Epub 2013/11/12. 10.1177/1087054713510562 . 24214970

[126] Grosso G, Galvano F, Marventano S, Malaguarnera M, Bucolo C, Drago F, et al Omega-3 fatty acids and depression: scientific evidence and biological mechanisms. Oxidative medicine and cellular longevity. 2014;2014:313570 Epub 2014/04/24. 10.1155/2014/313570 24757497 PMC3976923

[127] Grosso G, Pajak A, Marventano S, Castellano S, Galvano F, Bucolo C, et al Role of omega-3 Fatty acids in the treatment of depressive disorders: a comprehensive meta-analysis of randomized clinical trials. PloS one. 2014;9(5):e96905 Epub 2014/05/09. 10.1371/journal.pone.0096905 24805797 PMC4013121

[128] Hammamieh R, Chakraborty N, Gautam A, Miller SA, Muhie S, Meyerhoff J, et al Transcriptomic analysis of the effects of a fish oil enriched diet on murine brains. PloS one. 2014;9(3):e90425 Epub 2014/03/19. 10.1371/journal.pone.0090425 24632812 PMC3954562

[129] Zugno AI, Chipindo HL, Volpato AM, Budni J, Steckert AV, de Oliveira MB, et al Omega-3 prevents behavior response and brain oxidative damage in the ketamine model of schizophrenia. Neuroscience. 2014;259:223–31. Epub 2013/12/10. 10.1016/j.neuroscience.2013.11.049 . 24316471

[130] Fedorova I, Hussein N, Baumann MH, Di Martino C, Salem N Jr. An n-3 fatty acid deficiency impairs rat spatial learning in the Barnes maze. Behavioral neuroscience. 2009;123(1):196–205. Epub 2009/01/28. 10.1037/a0013801 . 19170444

[131] Chalon S. Omega-3 fatty acids and monoamine neurotransmission. Prostaglandins, leukotrienes, and essential fatty acids. 2006;75(4–5):259–69. Epub 2006/09/12. 10.1016/j.plefa.2006.07.005 . 16963244

[132] Bourre JM, Pascal G, Durand G, Masson M, Dumont O, Piciotti M. Alterations in the fatty acid composition of rat brain cells (neurons, astrocytes, and oligodendrocytes) and of subcellular fractions (myelin and synaptosomes) induced by a diet devoid of n-3 fatty acids. Journal of neurochemistry. 1984;43(2):342–8. Epub 1984/08/01. . 6736955 10.1111/j.1471-4159.1984.tb00906.x

[133] Moriguchi T, Salem N Jr. Recovery of brain docosahexaenoate leads to recovery of spatial task performance. Journal of neurochemistry. 2003;87(2):297–309. Epub 2003/09/27. . 14511107 10.1046/j.1471-4159.2003.01966.x

[134] Levant B, Radel JD, Carlson SE. Decreased brain docosahexaenoic acid during development alters dopamine-related behaviors in adult rats that are differentially affected by dietary remediation. Behavioural brain research. 2004;152(1):49–57. Epub 2004/05/12. 10.1016/j.bbr.2003.09.029 . 15135968

[135] Chuang JC, Cui H, Mason BL, Mahgoub M, Bookout AL, Yu HG, et al Chronic social defeat stress disrupts regulation of lipid synthesis. Journal of lipid research. 2010;51(6):1344–53. Epub 2010/02/05. 10.1194/jlr.M002196 20129912 PMC3035497

[136] Gautam A, D'Arpa P, Donohue DE, Muhie S, Chakraborty N, Luke BT, et al Acute and chronic plasma metabolomic and liver transcriptomic stress effects in a mouse model with features of post-traumatic stress disorder. PloS one. 2015;10(1):e0117092 Epub 2015/01/30. 10.1371/journal.pone.0117092 . 25629821 PMC4309402

[137] Muskiet FAJ. Pathophysiology and Evolutionary Aspects of Dietary Fats and Long-Chain Polyunsaturated Fatty Acids across the Life Cycle In: Montmayeur JP, le Coutre J, editors. Fat Detection: Taste, Texture, and Post Ingestive Effects. Frontiers in Neuroscience Boca Raton (FL) 2010. 21452482

[138] Matsumura K, Noguchi H, Nishi D, Matsuoka Y. The effect of omega-3 fatty acids on psychophysiological assessment for the secondary prevention of posttraumatic stress disorder: an open-label pilot study. Global journal of health science. 2012;4(1):3–9. Epub 2012/09/18. 10.5539/gjhs.v4n1p3 . 22980098 PMC4777038

[139] Matsuoka Y, Nishi D, Yonemoto N, Hamazaki K, Matsumura K, Noguchi H, et al Tachikawa project for prevention of posttraumatic stress disorder with polyunsaturated fatty acid (TPOP): study protocol for a randomized controlled trial. BMC psychiatry. 2013;13:8 Epub 2013/01/08. 10.1186/1471-244X-13-8 23289548 PMC3598223

[140] Rodrigues AF, Roecker R, Junges GM, de Lima DD, da Cruz JG, Wyse AT, et al Hypoxanthine induces oxidative stress in kidney of rats: protective effect of vitamins E plus C and allopurinol. Cell Biochem Funct. 2014;32(4):387–94. 10.1002/cbf.3029 . 24578313

[141] Mesquita Casagrande AC, Wamser MN, de Lima DD, Pereira da Cruz JG, Wyse AT, Dal Magro DD. In vitro stimulation of oxidative stress by hypoxanthine in blood of rats: prevention by vitamins e plus C and allopurinol. Nucleosides Nucleotides Nucleic Acids. 2013;32(1):42–57. 10.1080/15257770.2012.760043 . 23360294

[142] Puurunen J, Tiira K, Lehtonen M, Hanhineva K, Lohi H. Non-targeted metabolite profiling reveals changes in oxidative stress, tryptophan and lipid metabolisms in fearful dogs. Behav Brain Funct. 2016;12(1):7 10.1186/s12993-016-0091-2 26867941 PMC4751666

[143] Su ZH, Li SQ, Zou GA, Yu CY, Sun YG, Zhang HW, et al Urinary metabonomics study of anti-depressive effect of Chaihu-Shu-Gan-San on an experimental model of depression induced by chronic variable stress in rats. J Pharm Biomed Anal. 2011;55(3):533–9. 10.1016/j.jpba.2011.02.013 . 21398066

[144] Chang SJ, Chen SM, Chiang SL, Chang KL, Ko YC. Association between cigarette smoking and hypoxanthine guanine phosphoribosyltransferase activity. Kaohsiung J Med Sci. 2005;21(11):495–501. 10.1016/S1607-551X(09)70157-3 . 16358551 PMC11917623

[145] Yamamoto T, Moriwaki Y, Takahashi S. Effect of ethanol on metabolism of purine bases (hypoxanthine, xanthine, and uric acid). Clin Chim Acta. 2005;356(1–2):35–57. 10.1016/j.cccn.2005.01.024 . 15936302

[146] Ryu HM, Kim YJ, Oh EJ, Oh SH, Choi JY, Cho JH, et al Hypoxanthine induces cholesterol accumulation and incites atherosclerosis in apolipoprotein E-deficient mice and cells. J Cell Mol Med. 2016 10.1111/jcmm.12916 . 27396856 PMC5082407

[147] Lewis GD, Wei R, Liu E, Yang E, Shi X, Martinovic M, et al Metabolite profiling of blood from individuals undergoing planned myocardial infarction reveals early markers of myocardial injury. The Journal of clinical investigation. 2008;118(10):3503–12. 10.1172/JCI35111 18769631 PMC2525696

[148] Moseley HN. Error Analysis and Propagation in Metabolomics Data Analysis. Comput Struct Biotechnol J. 2013;4(5). 10.5936/csbj.201301006 23667718 PMC3647477

[149] Tzoulaki I, Ebbels TM, Valdes A, Elliott P, Ioannidis JP. Design and analysis of metabolomics studies in epidemiologic research: a primer on -omic technologies. Am J Epidemiol. 2014;180(2):129–39. 10.1093/aje/kwu143 . 24966222

[150] Li SH, Graham BM. Why are women so vulnerable to anxiety, trauma-related and stress-related disorders? The potential role of sex hormones. Lancet Psychiatry. 2017;4(1):73–82. 10.1016/S2215-0366(16)30358-3 . 27856395

[151] Shah SH, Kraus WE, Newgard CB. Metabolomic profiling for the identification of novel biomarkers and mechanisms related to common cardiovascular diseases: form and function. Circulation. 2012;126(9):1110–20. Epub 2012/08/29. 10.1161/CIRCULATIONAHA.111.060368 . 22927473 PMC4374548

[152] Mapstone M, Cheema AK, Fiandaca MS, Zhong X, Mhyre TR, Macarthur LH, et al Plasma phospholipids identify antecedent memory impairment in older adults. Nature medicine. 2014;20(4):415–8. 10.1038/nm.3466 24608097 PMC5360460

[153] Noga MJ, Dane A, Shi S, Attali A, van Aken H, Suidgeest E, et al Metabolomics of cerebrospinal fluid reveals changes in the central nervous system metabolism in a rat model of multiple sclerosis. Metabolomics: Official journal of the Metabolomic Society. 2012;8(2):253–63. Epub 2012/03/27. 10.1007/s11306-011-0306-3 22448154 PMC3291832

[154] Cuthbert BN, Insel TR. Toward the future of psychiatric diagnosis: the seven pillars of RDoC. BMC medicine. 2013;11:126 10.1186/1741-7015-11-126 23672542 PMC3653747

[155] Stein JY, Wilmot DV, Solomon Z. Does one size fit all? Nosological, clinical, and scientific implications of variations in PTSD Criterion A. J Anxiety Disord. 2016;43:106–17. 10.1016/j.janxdis.2016.07.001 . 27449856

[156] Valero T. Mitochondrial biogenesis: pharmacological approaches. Current pharmaceutical design. 2014;20(35):5507–9. . 24606795 10.2174/138161282035140911142118

[157] Chiang MC, Chern Y, Huang RN. PPARgamma rescue of the mitochondrial dysfunction in Huntington's disease. Neurobiology of disease. 2012;45(1):322–8. Epub 2011/09/13. 10.1016/j.nbd.2011.08.016 . 21907283

[158] Mortiboys H, Aasly J, Bandmann O. Ursocholanic acid rescues mitochondrial function in common forms of familial Parkinson's disease. Brain: a journal of neurology. 2013;136(Pt 10):3038–50. Epub 2013/09/04. 10.1093/brain/awt224 . 24000005

[159] Valaasani KR, Sun Q, Hu G, Li J, Du F, Guo Y, et al Identification of human ABAD inhibitors for rescuing Abeta-mediated mitochondrial dysfunction. Current Alzheimer research. 2014;11(2):128–36. Epub 2014/02/01. 24479630 10.2174/1567205011666140130150108 PMC4082996

[160] Chen WW, Birsoy K, Mihaylova MM, Snitkin H, Stasinski I, Yucel B, et al Inhibition of ATPIF1 ameliorates severe mitochondrial respiratory chain dysfunction in mammalian cells. Cell reports. 2014;7(1):27–34. Epub 2014/04/02. 10.1016/j.celrep.2014.02.046 24685140 PMC4040975

[161] Ipser JC, Stein DJ. Evidence-based pharmacotherapy of post-traumatic stress disorder (PTSD). Int J Neuropsychopharmacol. 2012;15(6):825–40. 10.1017/S1461145711001209 . 21798109

