# Supplementary material for: Metabolomic analysis of male combat veterans with post traumatic stress disorder
Source: PLoS One. 2019 Mar 18;14(3):e0213839. doi: 10.1371/journal.pone.0213839 (PMC6422302; doi:10.1371/journal.pone.0213839)
Supplement: S1 File — Table A. Analytes identified in the discovery group that were not significantly different between PTSD positive and PTSD negative subjects Figure A. Barplot of metabolite validation success fraction over 1000 permutations of discovery and test groups. (DOCX) [file pone.0213839.s001.docx]

**Supporting Information:**

**Sample Preparation and Metabolic Profiling**

Metabolic profiling of all plasma samples was performed at Metabolon, Inc. (Durham, NC). The metabolic profiling used three independent platforms: ultrahigh performance liquid chromatography/tandem mass spectrometry (UHPLC/MS/MS^2^) optimized for basic species, UHPLC/MS/MS^2^ optimized for acidic species, and gas chromatography/mass spectrometry (GC/MS). Samples were processed as described [1, 2]. For each sample, 100μL of plasma was used for analyses. Using an automated liquid handler (Hamilton LabStar, Salt Lake City, UT), protein was precipitated from the plasma with methanol that contained four standards, used to determine extraction efficiency. The resulting supernatant was split into equal aliquots for analysis on the three platforms. Aliquots, dried under nitrogen and vacuum-desiccated, were subsequently either reconstituted in 50 μL 0.1% formic acid in water (acidic conditions) or in 50 μL 6.5 mM ammonium bicarbonate in water, pH 8 (basic conditions) for the two UHPLC/MS/MS^2^ analyses, or were derivatized at 60^o^C for one hour using equal parts bistrimethyl-silyl-trifluoroacetamide and solvent mixture acetonitrile:dichloromethane: cyclohexane (5:4:1) with 5% triethylamine, to a final volume of 50 μL for GC/MS analysis. In addition, three types of controls were analyzed in concert with the experimental samples: aliquots of a well-characterized human plasma pool served as technical replicates throughout the data set, extracted water samples served as process blanks, and a cocktail of standards spiked into every analyzed sample allowed monitoring of instrument performance. Experimental PTSD samples and combat controls were randomized across platform run days.

For UHPLC/MS/MS^2^ analysis, aliquots were separated using a Waters Acquity UPLC (Waters, Millford, MA) and analyzed using an LTQ mass spectrometer (Thermo Fisher Scientific, Inc., Waltham, MA), which consisted of an electrospray ionization source and linear ion-trap mass analyzer. The MS instrument scanned 99-1000 *m/z* and alternated between MS and MS^2^ scans using dynamic exclusion with approximately 6 scans per second. Derivatized samples for GC/MS were separated on a 5% phenyldimethyl silicone column with helium as the carrier gas and a temperature ramp from 60°C to 340°C and then analyzed on a Thermo-Finnigan Trace DSQ MS (Thermo Fisher Scientific, Inc.) operated at unit mass resolving power with electron impact ionization and a 50-750 atomic mass unit scan range. Metabolites were identified by automated comparison of the ion features in the experimental samples to a reference library of chemical standard entries that included retention time, molecular weight (*m/z*), preferred adducts, in-source fragments, and associated MS spectra, and were curated by visual inspection for quality control using software developed at Metabolon [3].

Instrument variability was determined by calculating the median relative standard deviation for the internal standards that were added to each sample prior to injection into the mass spectrometers. Overall process variability was determined by calculating the median relative standard deviation for all endogenous metabolites (i.e., non-instrument standards) present in 100% of the matrix samples, which are technical replicates of pooled samples. Values for instrument and process variability were 6% and 12%, respectively. Discovery samples and test samples were run in separate batches, and 12 random discovery samples re-run with the test samples to serve as anchors for quantitation. Because the two metabolomic analyses used slightly different methodologies that resulted in detection and identification of some different compounds in each run, only compounds that were identified in both the discovery and test samples were used in the analysis.

| **S1 File Table A. Analytes identified in the discovery group that were not significantly different between PTSD positive and PTSD negative subjects** | | | | | | | | |
| --- | --- | --- | --- | --- | --- | --- | --- | --- |
| **Super Pathway** | **Sub Pathway** | **Metabolite** | **PTSD - (N=51)** | | **PTSD + (N=52)** | | **Sig. (P)** | **Q Value** |
|  |  |  | **Mean** | **St.Dev.** | **Mean** | **St.Dev.** |  |  |
| Amino acid | Glycine, serine and threonine metabolism | glycine | 1.06 | 0.26 | 0.95 | 0.27 | .056 | 0.386 |
|  |  | sarcosine (N-methylglycine) | 1.03 | 0.33 | 1.11 | 0.42 | .379 | 0.603 |
|  |  | dimethylglycine | 1.05 | 0.23 | 1.05 | 0.30 | .713 | 0.713 |
|  |  | N-acetylglycine | 1.27 | 1.24 | 1.00 | 0.54 | .055 | 0.386 |
|  |  | serine | 1.05 | 0.25 | 1.01 | 0.33 | .228 | 0.603 |
|  |  | threonine | 0.99 | 0.33 | 0.93 | 0.37 | .293 | 0.544 |
|  |  | N-acetylthreonine | 1.07 | 0.23 | 1.03 | 0.21 | .361 | 0.602 |
|  |  | betaine | 1.05 | 0.22 | 1.02 | 0.23 | .495 | 0.645 |
|  | Alanine and aspartate metabolism | asparagine | 1.01 | 0.25 | 0.95 | 0.26 | .304 | 0.549 |
|  |  | beta-alanine | 1.05 | 0.45 | 1.06 | 0.42 | .940 | 0.774 |
|  |  | N-acetyl-beta-alanine | 0.98 | 0.35 | 1.08 | 0.40 | .158 | 0.473 |
|  |  | alanine | 1.00 | 0.28 | 1.09 | 0.30 | .078 | 0.386 |
|  |  | N-acetylalanine | 1.02 | 0.25 | 1.03 | 0.28 | .957 | 0.774 |
|  | Glutamate metabolism | glutamate | 1.01 | 0.55 | 1.20 | 0.61 | .089 | 0.399 |
|  |  | pyroglutamine* | 1.09 | 0.62 | 1.16 | 0.67 | .608 | 0.695 |
|  | Histidine metabolism | histidine | 1.03 | 0.12 | 1.00 | 0.13 | .270 | 0.540 |
|  |  | 3-methylhistidine | 1.03 | 1.03 | 0.80 | 0.73 | .285 | 0.540 |
|  | Lysine metabolism | lysine | 1.05 | 0.20 | 1.01 | 0.16 | .388 | 0.603 |
|  |  | pipecolate | 1.71 | 1.65 | 1.44 | 1.60 | .409 | 0.604 |
|  |  | N6-acetyllysine | 1.07 | 0.25 | 1.03 | 0.16 | .713 | 0.713 |
|  |  | glutarylcarnitine-C5 | 1.03 | 0.29 | 1.09 | 0.33 | .279 | 0.540 |
|  | Phenylalanine & tyrosine metabolism | phenylalanine | 1.00 | 0.12 | 1.04 | 0.17 | .351 | 0.602 |
|  |  | p-cresol-sulfate | 1.05 | 0.63 | 0.99 | 0.61 | .438 | 0.604 |
|  |  | tyrosine | 1.02 | 0.17 | 1.11 | 0.29 | .076 | 0.386 |
|  |  | 3-(4-hydroxyphenyl)lactate | 1.01 | 0.26 | 1.08 | 0.35 | .385 | 0.603 |
|  |  | 3-methoxytyrosine | 1.05 | 0.25 | 0.99 | 0.24 | .164 | 0.473 |
|  |  | phenylacetylglutamine | 1.03 | 0.57 | 1.13 | 0.58 | .604 | 0.694 |
|  |  | phenol sulfate | 1.53 | 2.18 | 1.31 | 0.79 | .805 | 0.738 |
|  | Tryptophan metabolism | kynurenine | 0.96 | 0.20 | 1.05 | 0.24 | .070 | 0.386 |
|  |  | tryptophan | 1.00 | 0.14 | 1.04 | 0.16 | .336 | 0.595 |
|  |  | indolelactate | 1.05 | 0.32 | 1.16 | 0.38 | .115 | 0.434 |
|  |  | indoleacetate | 1.11 | 0.45 | 1.20 | 0.60 | .974 | 0.774 |
|  |  | tryptophan-betaine | 1.70 | 1.68 | 1.78 | 2.15 | .737 | 0.722 |
|  |  | serotonin | 1.61 | 1.45 | 1.43 | 2.42 | .189 | 0.487 |
|  |  | C-glycosyltryptophan | 1.01 | 0.20 | 1.04 | 0.21 | .689 | 0.713 |
|  |  | 3-indoxylsulfate | 1.06 | 0.37 | 1.05 | 0.34 | .948 | 0.774 |
|  |  | indolepropionate | 1.14 | 0.78 | 1.02 | 0.50 | .353 | 0.602 |
|  | Valine, leucine and isoleucine metabolism | 3-methyl-2-oxobutyrate | 1.04 | 0.21 | 1.05 | 0.25 | .967 | 0.774 |
|  |  | 3-methyl-2-oxovalerate | 1.01 | 0.22 | 1.08 | 0.28 | .266 | 0.540 |
|  |  | betahydroxyisovalerate | 1.15 | 0.41 | 1.07 | 0.37 | .351 | 0.602 |
|  |  | isoleucine | 1.02 | 0.25 | 1.04 | 0.19 | .391 | 0.603 |
|  |  | leucine | 1.03 | 0.23 | 1.05 | 0.18 | .410 | 0.604 |
|  |  | valine | 1.02 | 0.23 | 1.03 | 0.18 | .548 | 0.665 |
|  |  | 4-methyl-2-oxopentanoate | 1.01 | 0.21 | 1.08 | 0.28 | .216 | 0.521 |
|  |  | alpha-hydroxyisovalerate | 1.17 | 0.67 | 1.50 | 1.01 | .077 | 0.386 |
|  |  | isobutyrylcarnitine | 1.09 | 0.49 | 1.00 | 0.41 | .435 | 0.604 |
|  |  | 2-methylbutyrylcarnitine-C5 | 1.14 | 0.50 | 1.10 | 0.34 | .810 | 0.738 |
|  |  | isovalerylcarnitine | 1.09 | 0.48 | 1.06 | 0.37 | .963 | 0.774 |
|  |  | tiglylcarnitine | 1.00 | 0.37 | 0.95 | 0.36 | .519 | 0.651 |
|  | Cysteine, methionine, SAM, taurine metabolism | cysteine | 0.97 | 0.21 | 1.03 | 0.21 | .142 | 0.463 |
|  |  | S-methylcysteine | 1.28 | 1.09 | 1.07 | 0.73 | .621 | 0.699 |
|  |  | cystine | 1.02 | 0.39 | 1.11 | 0.34 | .112 | 0.433 |
|  |  | N-formylmethionine | 1.02 | 0.23 | 1.02 | 0.26 | .995 | 0.781 |
|  |  | methionine | 1.05 | 0.22 | 1.03 | 0.20 | .580 | 0.683 |
|  |  | N-acetylmethionine | 0.70 | 0.37 | 0.83 | 0.47 | .074 | 0.386 |
|  |  | alpha-ketobutyrate | 1.12 | 0.52 | 1.08 | 1.00 | .195 | 0.495 |
|  | Urea cycle; arginine-, proline-, metabolism | dimethylarginine-SDMA (ADMA) | 1.05 | 0.23 | 1.01 | 0.18 | .400 | 0.603 |
|  |  | ornithine | 1.07 | 0.44 | 1.21 | 0.47 | .132 | 0.447 |
|  |  | urea | 1.10 | 0.33 | 1.00 | 0.30 | .144 | 0.463 |
|  |  | proline | 1.07 | 0.24 | 1.08 | 0.32 | .661 | 0.713 |
|  |  | citrulline | 1.03 | 0.23 | 0.99 | 0.24 | .430 | 0.604 |
|  |  | N-methylproline | 2.16 | 2.77 | 2.04 | 3.01 | .540 | 0.660 |
|  |  | trans-4-hydroxyproline | 1.25 | 0.83 | 1.18 | 0.95 | .274 | 0.540 |
|  | Creatine metabolism | creatine | 1.07 | 0.43 | 1.13 | 0.52 | .644 | 0.711 |
|  |  | creatinine_m | 1.03 | 0.20 | 0.99 | 0.16 | .358 | 0.602 |
|  | Butanoate metabolism | 2-aminobutyrate | 1.01 | 0.30 | 0.98 | 0.28 | .673 | 0.713 |
|  | Glutathione metabolism | cysteine-glutathione-disulfide | 0.87 | 0.52 | 0.79 | 0.54 | .438 | 0.604 |
| Peptide | Dipeptide | pro-hydroxy-pro | 1.23 | 0.89 | 1.03 | 0.39 | .523 | 0.652 |
|  | gamma-glutamyl | gamma-glutamylvaline | 1.10 | 0.32 | 1.01 | 0.23 | .161 | 0.473 |
|  |  | gamma-glutamylleucine | 1.10 | 0.47 | 1.07 | 0.25 | .411 | 0.604 |
|  |  | gamma-glutamylisoleucine* | 1.15 | 0.54 | 1.05 | 0.29 | .712 | 0.713 |
|  |  | gamma-glutamylmethionine | 1.07 | 0.38 | 1.00 | 0.32 | .419 | 0.604 |
|  |  | gamma-glutamylglutamine | 1.05 | 0.29 | 1.02 | 0.26 | .600 | 0.694 |
|  |  | gamma-glutamylphenylalanine | 1.03 | 0.23 | 1.08 | 0.23 | .279 | 0.540 |
|  |  | gamma-glutamyltyrosine | 1.05 | 0.26 | 1.11 | 0.23 | .058 | 0.386 |
|  |  | gamma-glutamylthreonine | 0.84 | 0.43 | 0.72 | 0.36 | .111 | 0.433 |
| Carbohydrate | Aminosugars metabolism | erythronate* | 1.04 | 0.23 | 1.05 | 0.28 | .671 | 0.713 |
|  | Fructose, mannose, galactose, starch, and sucrose metabolism | fructose | 1.01 | 0.21 | 1.12 | 0.63 | .780 | 0.738 |
|  |  | maltose | 0.87 | 0.88 | 0.94 | 1.41 | .822 | 0.738 |
|  |  | mannose | 1.04 | 0.23 | 1.08 | 0.29 | .479 | 0.633 |
|  |  | Maltotriose | 0.68 | 0.90 | 0.75 | 1.21 | .855 | 0.745 |
|  | Glycolysis, gluconeogenesis, pyruvate metabolism | 1,5- anhydroglucitol (1,5-AG) | 1.00 | 0.30 | 1.01 | 0.30 | .986 | 0.780 |
|  |  | glycerate | 1.01 | 0.27 | 0.95 | 0.31 | .238 | 0.523 |
|  |  | glucose_m | 1.00 | 0.11 | 1.05 | 0.22 | .223 | 0.523 |
|  |  | glucuronate | 1.02 | 0.35 | 1.02 | 0.50 | .580 | 0.683 |
|  | Nucleotide sugars, pentose metabolism | gluconate | 1.04 | 0.33 | 1.04 | 0.43 | .810 | 0.738 |
| Energy | Krebs cycle | alpha-ketoglutarate | 1.08 | 0.46 | 1.15 | 0.48 | .400 | 0.604 |
|  |  | succinate | 1.00 | 0.25 | 1.06 | 0.28 | .285 | 0.540 |
|  |  | malate | 0.98 | 0.32 | 1.06 | 0.32 | .207 | 0.519 |
|  | Oxidative phosphorylation | phosphate | 1.01 | 0.17 | 0.98 | 0.12 | .635 | 0.705 |
| Lipid | Essential fatty acid | linoleate (18:2n6) | 1.07 | 0.36 | 0.97 | 0.38 | .130 | 0.447 |
|  |  | eicosapentaenoate (EPA; 20:5n3) | 1.13 | 0.59 | 1.05 | 0.58 | .368 | 0.602 |
|  |  | docosapentaenoate (n3-DPA; 22:5n3) | 1.16 | 0.61 | 1.00 | 0.64 | .083 | 0.391 |
|  |  | docosapentaenoate-n6-DPA-22-5n6 | 1.11 | 0.59 | 1.00 | 0.52 | .184 | 0.487 |
|  | Medium chain fatty acid | caproate (6:0) | 0.98 | 0.30 | 0.97 | 0.27 | .922 | 0.765 |
|  |  | heptanoate (7:0) | 0.96 | 0.40 | 0.95 | 0.32 | .826 | 0.738 |
|  |  | caprylate (8:0) | 1.03 | 0.40 | 1.00 | 0.34 | .833 | 0.739 |
|  |  | caprate (10:0) | 1.08 | 0.49 | 1.09 | 0.55 | .890 | 0.755 |
|  |  | 10-undecenoate-11-1n1 | 1.04 | 0.32 | 0.96 | 0.39 | .104 | 0.427 |
|  |  | laurate (12:0) | 1.16 | 0.87 | 1.12 | 0.48 | .812 | 0.738 |
|  | Long chain fatty acid | myristate (14:0) | 1.10 | 0.45 | 1.07 | 0.49 | .623 | 0.699 |
|  |  | myristoleate (14:1n5) | 1.21 | 0.61 | 1.09 | 0.66 | .216 | 0.521 |
|  |  | pentadecanoate (15:0) | 1.04 | 0.26 | 1.06 | 0.33 | .967 | 0.774 |
|  |  | palmitate (16:0) | 1.03 | 0.30 | 1.01 | 0.33 | .712 | 0.713 |
|  |  | palmitoleate (16:1n7) | 1.20 | 0.64 | 1.04 | 0.54 | .236 | 0.523 |
|  |  | margarate (17:0) | 1.14 | 0.41 | 1.09 | 0.60 | .277 | 0.540 |
|  |  | 10-heptaadecenoate (17:1n7) | 1.17 | 0.56 | 1.03 | 0.54 | .122 | 0.440 |
|  |  | stearate (18:0) | 1.04 | 0.26 | 0.98 | 0.33 | .161 | 0.473 |
|  |  | nonadecanoate (19:0) | 1.09 | 0.33 | 1.01 | 0.46 | .065 | 0.386 |
|  |  | arachidate-20-0 | 0.89 | 0.40 | 0.91 | 0.36 | .913 | 0.763 |
|  |  | mead acid-20-3n9 | 1.03 | 0.43 | 0.97 | 0.52 | .298 | 0.545 |
|  |  | arachidonate (20:4n6) | 1.03 | 0.38 | 0.98 | 0.33 | .420 | 0.604 |
|  |  | docosadienoate-22-2n6 | 1.07 | 0.41 | 0.94 | 0.41 | .095 | 0.399 |
|  |  | adrenate (22:4n6) | 1.01 | 0.37 | 1.01 | 0.42 | .958 | 0.774 |
|  | Fatty acid, monohydroxy | 2-hydroxydecanoate | 1.06 | 0.45 | 1.52 | 1.50 | .168 | 0.477 |
|  |  | 3-hydroxydecanoate | 0.91 | 0.45 | 1.06 | 0.52 | .156 | 0.473 |
|  |  | hydroxystearate | 1.04 | 0.25 | 1.01 | 0.26 | .472 | 0.631 |
|  |  | 2-hydroxypalmitate | 1.01 | 0.19 | 0.98 | 0.20 | .366 | 0.602 |
|  | Fatty acid, dicarboxylate | 2-hydroxyglutarate | 0.87 | 0.51 | 1.03 | 0.50 | .117 | 0.434 |
|  |  | hexadecanedioate | 1.05 | 0.56 | 1.62 | 4.61 | .805 | 0.738 |
|  |  | octadecanedioate | 1.06 | 0.57 | 1.25 | 1.20 | .702 | 0.713 |
|  |  | 3-carboxy-4-methyl-5-propyl-2-furanpropanoate (CMPF) | 1.82 | 2.35 | 1.88 | 2.51 | .442 | 0.605 |
|  | Fatty acid, branched | 13-methylmyristate | 1.03 | 0.30 | 0.99 | 0.36 | .299 | 0.545 |
|  |  | 15-methylpalmitate | 1.11 | 0.39 | 1.05 | 0.49 | .241 | 0.523 |
|  |  | 17-methylstearate | 1.14 | 0.46 | 1.01 | 0.62 | .067 | 0.386 |
|  | Fatty acid metabolism (also BCAA metabolism) | propionylcarnitine | 1.04 | 0.33 | 1.05 | 0.31 | .784 | 0.738 |
|  |  | butyrylcarnitine | 1.36 | 1.01 | 1.30 | 0.76 | .892 | 0.755 |
|  | Fatty acid metabolism | Isovalerate | 0.86 | 0.27 | 0.87 | 0.33 | .923 | 0.765 |
|  | Carnitine metabolism | deoxycarnitine | 1.03 | 0.49 | 1.02 | 0.28 | .612 | 0.695 |
|  |  | carnitine | 0.98 | 0.13 | 1.03 | 0.15 | .071 | 0.386 |
|  |  | acetylcarnitine | 1.05 | 0.28 | 1.06 | 0.32 | .793 | 0.738 |
|  |  | laurylcarnitine | 1.09 | 0.55 | 1.22 | 0.56 | .132 | 0.447 |
|  |  | palmitoylcarnitine | 1.04 | 0.48 | 1.07 | 0.27 | .164 | 0.473 |
|  |  | stearoylcarnitine | 0.99 | 0.30 | 1.06 | 0.33 | .369 | 0.602 |
|  | Bile acid metabolism | cholate | 3.00 | 6.35 | 2.41 | 3.84 | .819 | 0.738 |
|  |  | glycocholate | 1.53 | 1.91 | 1.88 | 2.20 | .109 | 0.433 |
|  |  | ursodeoxycholate | 1.65 | 2.76 | 1.47 | 1.48 | .501 | 0.645 |
|  |  | deoxycholate | 0.77 | 0.98 | 0.92 | 0.89 | .229 | 0.523 |
|  |  | glycochenodeoxycholate | 1.22 | 0.91 | 1.37 | 0.99 | .186 | 0.487 |
|  |  | glycolithocholate-sulfate | 1.19 | 0.83 | 1.17 | 0.97 | .765 | 0.735 |
|  |  | taurolithocholate-3-sulfate | 0.84 | 0.65 | 0.70 | 0.63 | .231 | 0.523 |
|  |  | glycodeoxycholate | 1.43 | 1.48 | 1.93 | 2.26 | .091 | 0.399 |
|  |  | glycocholenate-sulfate | 1.13 | 0.45 | 1.06 | 0.43 | .437 | 0.604 |
|  |  | glycoursodeoxycholate | 1.51 | 2.02 | 1.72 | 2.25 | .679 | 0.713 |
|  | Glycerolipid metabolism | glycerol | 1.03 | 0.25 | 1.01 | 0.26 | .858 | 0.745 |
|  |  | choline | 1.01 | 0.16 | 1.02 | 0.21 | .887 | 0.755 |
|  |  | Glycerol 3-phosphate (G3P) | 0.96 | 0.28 | 1.07 | 0.36 | .123 | 0.440 |
|  |  | glycerophosphorylcholine (GPC) | 0.98 | 0.32 | 1.05 | 0.54 | .990 | 0.780 |
|  | Inositol metabolism | myo-inositol | 1.02 | 0.20 | 1.01 | 0.21 | .681 | 0.713 |
|  | Ketone bodies | 3-hydroxybutyrate (BHBA) | 1.86 | 2.48 | 1.25 | 1.39 | .060 | 0.386 |
|  | Lysolipid | 1-palmitoyl-GPE-16-0 | 0.98 | 0.36 | 1.05 | 0.34 | .183 | 0.487 |
|  |  | 1-stearoyl-GPE-18-0 | 1.02 | 0.24 | 1.06 | 0.32 | .699 | 0.713 |
|  |  | 1-oleoyl-GPE-18-1 | 1.08 | 0.40 | 1.20 | 0.49 | .251 | 0.538 |
|  |  | 1-linoleoyl-GPE-18-2 | 1.06 | 0.45 | 1.11 | 0.54 | .701 | 0.713 |
|  |  | 1-arachidonoyl-GPE-20-4 | 1.11 | 0.42 | 1.13 | 0.55 | .973 | 0.774 |
|  |  | 1-palmitoyl (GPC) 16-0 | 1.03 | 0.21 | 1.03 | 0.16 | .561 | 0.677 |
|  |  | 2-palmitoyl (GPC) 16-0 | 1.04 | 0.38 | 1.05 | 0.31 | .679 | 0.713 |
|  |  | 1-palmitoleoyl (GPC) 16-1 | 1.07 | 0.36 | 1.10 | 0.35 | .505 | 0.645 |
|  |  | 2-palmitoleoyl (GPC) 16-1 | 0.98 | 0.47 | 0.99 | 0.36 | .628 | 0.701 |
|  |  | 1-stearoyl (GPC) 18-0 | 1.24 | 0.69 | 1.16 | 0.68 | .346 | 0.602 |
|  |  | 1-oleoyl (GPC) 18-1 | 1.01 | 0.29 | 0.99 | 0.25 | .784 | 0.738 |
|  |  | 1-linoleoyl (GPC) 18-2 | 1.03 | 0.24 | 1.01 | 0.19 | .600 | 0.694 |
|  |  | 1-arachidonoyl (GPC) 20-4 | 0.98 | 0.31 | 1.04 | 0.27 | .223 | 0.523 |
|  |  | 1-palmitoyl-GPI-16-0 | 0.78 | 0.56 | 0.79 | 0.47 | .913 | 0.763 |
|  |  | 1-stearoyl-GPI-18-0 | 1.03 | 0.42 | 1.11 | 0.42 | .387 | 0.603 |
|  |  | 1-oleoyl-GPI-18-1 | 1.03 | 0.75 | 0.91 | 0.57 | .707 | 0.713 |
|  |  | 1arachidonoylGPI204 | 0.99 | 0.36 | 1.04 | 0.36 | .420 | 0.604 |
|  | Sphingolipid | sphingosine | 0.99 | 0.74 | 1.15 | 0.95 | .321 | 0.574 |
|  |  | palmitoyl-sphingomyelin-d18-1-16-0 | 1.00 | 0.19 | 1.00 | 0.23 | .880 | 0.755 |
|  |  | stearoyl-sphingomyelin-d18-1-18-0_A | 1.01 | 0.25 | 1.09 | 0.40 | .400 | 0.603 |
|  | Sterol/Steroid | cholesterol_m | 1.02 | 0.19 | 1.02 | 0.20 | .834 | 0.739 |
|  |  | dehydroisoandrosteronesulfate (DHEAS) | 0.98 | 0.42 | 1.02 | 0.54 | .899 | 0.758 |
|  |  | epiandrosterone sulfate | 1.10 | 0.55 | 0.95 | 0.51 | .152 | 0.473 |
|  |  | androsterone sulfate | 1.13 | 0.62 | 0.99 | 0.61 | .186 | 0.487 |
|  |  | cortisone | 0.94 | 0.28 | 1.05 | 0.28 | .071 | 0.386 |
|  |  | 7-alpha-hydroxy-3-oxo-4-cholestenoate-7-Hoca | 1.00 | 0.37 | 1.11 | 0.46 | .171 | 0.479 |
|  |  | 4-androsten-3-beta-17-beta-diol-disulfate-1 | 0.99 | 0.60 | 1.41 | 1.12 | .068 | 0.386 |
|  |  | 4-androsten-3-beta-17-beta-diol-disulfate-2 | 1.04 | 0.43 | 1.10 | 0.44 | .491 | 0.645 |
|  |  | 5-alpha-androstan-3-beta-17-beta-diol-disulfate | 1.00 | 0.62 | 1.25 | 0.93 | .381 | 0.603 |
|  |  | pregnen-diol-disulfate | 1.12 | 0.61 | 1.27 | 0.83 | .500 | 0.645 |
|  |  | pregn-steroid-monosulfate | 1.01 | 0.47 | 1.00 | 0.57 | .759 | 0.735 |
|  |  | andro-steroid-monosulfate-1 | 1.15 | 0.78 | 1.23 | 0.80 | .734 | 0.722 |
|  |  | 21-hydroxypregnenolone-disulfate | 0.86 | 0.43 | 0.93 | 0.51 | .530 | 0.652 |
|  |  | pregnenolonesulfate | 1.05 | 0.48 | 1.19 | 0.77 | .595 | 0.694 |
|  |  | pregnanediol-3-glucuronide | 0.90 | 0.49 | 0.90 | 0.58 | .956 | 0.774 |
| Nucleotide | Purine metabolism, (hypo)xanthine/inosine containing | xanthine | 0.95 | 0.35 | 0.99 | 0.42 | .824 | 0.738 |
|  | Purine metabolism, adenine containing | N1-methyladenosine | 1.01 | 0.23 | 1.02 | 0.15 | .262 | 0.540 |
|  |  | adenosine5monophosphate (AMP) | 1.28 | 0.86 | 1.41 | 1.58 | .860 | 0.745 |
|  | Purine metabolism, guanine containing | 7-methylguanine | 1.04 | 0.47 | 0.96 | 0.45 | .371 | 0.602 |
|  |  | N2,N2-dimethylguanosine | 1.07 | 0.53 | 1.07 | 0.37 | .732 | 0.722 |
|  |  | N6-carbamoylthreonyladenosine | 1.05 | 0.35 | 1.02 | 0.23 | .845 | 0.742 |
|  | Purine metabolism, urate metabolism | urate | 1.00 | 0.13 | 0.98 | 0.15 | .474 | 0.631 |
|  |  | allantoin | 0.99 | 0.50 | 1.08 | 0.43 | .188 | 0.487 |
|  | Pyrimidine metabolism, thymine containing; Valine, leucine and isoleucine metabolism | 3-aminoisobutyrate | 0.92 | 0.57 | 1.05 | 1.16 | .806 | 0.738 |
|  | Pyrimidine metabolism, uracil containing | uridine | 1.02 | 0.22 | 0.95 | 0.22 | .092 | 0.399 |
|  |  | pseudouridine | 1.00 | 0.18 | 1.02 | 0.14 | .271 | 0.540 |
| Cofactors and vitamins | Ascorbate and aldarate metabolism | threonate | 1.05 | 0.39 | 0.93 | 0.43 | .094 | 0.399 |
|  | Hemoglobin and porphyrin metabolism | bilirubin*Z-Z | 1.12 | 0.55 | 1.04 | 0.49 | .459 | 0.624 |
|  |  | bilirubin*E-E | 1.21 | 0.62 | 1.01 | 0.50 | .074 | 0.386 |
|  |  | biliverdin | 1.05 | 0.45 | 1.00 | 0.34 | .761 | 0.735 |
|  | Nicotinate and nicotinamide metabolism | nicotinamide | 1.12 | 0.64 | 1.31 | 1.01 | .579 | 0.683 |
|  |  | trigonelline (N-methylnicotinate) | 1.08 | 0.99 | 1.77 | 2.33 | .530 | 0.652 |
|  | Pantothenate and CoA metabolism | pantothenate | 1.21 | 0.69 | 1.11 | 0.64 | .508 | 0.645 |
|  | Tocopherol metabolism | alpha-tocopherol | 1.03 | 0.26 | 1.07 | 0.27 | .462 | 0.624 |
|  | Vitamin B6 metabolism | pyridoxate | 1.67 | 1.88 | 2.29 | 4.19 | .754 | 0.735 |
| Xenobioltics | Benzoate metabolism | hippurate | 1.21 | 1.01 | 1.35 | 0.94 | .283 | 0.540 |
|  |  | 3-methyl catechol sulfate 1 | 0.95 | 1.11 | 1.37 | 2.15 | .213 | 0.521 |
|  |  | 4-methylcatechol-sulfate | 1.31 | 1.01 | 1.45 | 1.84 | .823 | 0.738 |
|  |  | catechol-sulfate | 1.13 | 0.59 | 1.20 | 0.66 | .517 | 0.651 |
|  |  | benzoate | 1.02 | 0.21 | 0.96 | 0.20 | .136 | 0.453 |
|  |  | 4-vinylphenol sulfate | 1.47 | 1.83 | 1.61 | 1.33 | .082 | 0.391 |
|  |  | O-methylcatechol sulfate | 0.95 | 0.57 | 1.26 | 1.13 | .239 | 0.523 |
|  | Chemical | iminodiacetate-IDA | 1.01 | 0.25 | 1.06 | 0.24 | .293 | 0.544 |
|  | Drug | salicylate | 2.96 | 13.05 | 1.06 | 1.17 | .841 | 0.741 |
|  |  | 3-N-acetyl-L-cystein-Syl-acetaminophen | 0.17 | 0.27 | 0.14 | 0.12 | .566 | 0.679 |
|  | Food component/Plant | piperine | 1.41 | 1.36 | 1.47 | 1.68 | .679 | 0.713 |
|  |  | stachydrine | 1.72 | 1.81 | 1.41 | 1.63 | .709 | 0.713 |
|  |  | homostachydrine | 1.15 | 0.73 | 1.09 | 0.55 | .873 | 0.753 |
|  | Xanthine metabolism | caffeine | 1.68 | 2.09 | 3.09 | 6.41 | .735 | 0.722 |
|  |  | theobromine | 1.27 | 1.32 | 1.36 | 1.61 | .675 | 0.713 |
|  |  | 7-methylxanthine | 0.83 | 0.87 | 0.97 | 0.91 | .269 | 0.269 |
|  | Sugar, sugar substitute, starch | erythritol | 1.05 | 0.26 | 1.02 | 0.29 | .436 | 0.604 |

**Supporting Results**

We found several metabolites that were significantly different between PTSD-positive and PTSD–negative subjects in our discovery group, which were not replicated in our test group (Table 2). The lack of replication could be due to type I errors or due to small effect sizes and small numbers of subjects in our test group. Additional studies in larger cohorts are needed to confirm the that these metabolites are different between groups.

***Carnitines.*** To enter the mitochondria for ß-oxidation, fatty acids must be conjugated to carnitine and then to Coenzyme A. However, in subjects with PTSD, we found increases in three carnitines, octanoylcarnitine (p<0.004; q=0.13), hexanoylcarnitine (p<0.047; q=0.38), and decanoylcarnitine (p<0.003; q=0.12), suggesting there may be stalled entry of acyl chains into mitochondria for oxidation, and that fatty acids may not be used efficiently as a source of energy.

***Carbon-nitrogen balance and transaminase reactions.*** Several amino acids involved in transamination differed between control and PTSD subjects. Concentrations of glutamine (p<0.010; q=0.24) were lower in PTSD. Trans-urocanate (p<0.033; q=0.38), a transaminase product generated in the pathway from histidine to glutamate, was also less abundant in PTSD. These differences in amino acids may reflect changes in carbon-nitrogen balance and differences in contributions of amino acids to the TCA cycle. Because many of the enzymes in amino acid metabolism require cofactors (e.g., pyridoxal phosphate, flavin mononucleotide, nicotinamide adenine dinucleotide), the observed differences in amino acid metabolism may also reflect cofactor availability or altered mitochondrial function.

***Urea cycle.*** The urea cycle participates in carbon-nitrogen balance and in eliminating nitrogenous wastes generated by amino acid catabolism. In the liver, the urea cycle converts ammonia to urea, which is less toxic and more readily excreted; urea generation is connected to conversion of arginine to ornithine. Arginine concentrations were lower in PTSD (p<0.031; q=0.38), suggesting increased arginase activity, which generates urea from ammonia and converts arginine to ornithine. Arginase activity is regulated by glucose in rodent kidneys [4]; the higher glucose concentrations seen in PTSD (Table 1) may similarly contribute to increased arginase activity. Lactate also decreases intestinal synthesis of arginine from proline via an inhibition of proline oxidase[5]. Arginine also contributes to the TCA cycle by conversion to oxaloacetate, which may be diminished due to low arginine concentrations.

***Aromatic amino acids metabolites.*** Phenyllactate, a metabolite of the aromatic amino acid phenylalanine (generated by the gut microbiome), also differed between groups, and was greater in PTSD (p<0.026; q=0.36) suggesting that subjects with PTSD may differ in the composition and/or metabolic activity of the gut microbiome.

***Steroids.*** We identified 39 steroids in the plasma of our subjects (Table 2 and S1Table). The only one that was significantly different between groups was cortisol (p<0.012; q=0.25), which was increased in PTSD, consistent with our analysis of serum cortisol by ELISA (14.54 ± 7.62 µg/dl in PTSD compared to 11.64 ± 4.87 µg/dl in controls, p< 0.008).

***Branched Chain Amino Acids.*** Branched-chain amino acids (BCAA) can be catabolized to enter the TCA cycle for energy production. They have been implicated in the development of insulin resistance, with 3-hydroxyisobutyrate (a catabolic intermediate of valine) activating endothelial fatty acid transport, stimulating muscle fatty acid uptake and promoting lipid accumulation, leading to insulin resistance[6]. However, our PTSD-positive subjects show reduced 3-hydroxyisobutyrate (p<0.040; q=0.38), but increased insulin resistance.

***Sphingosine 1-phosphate.*** Sphingosine 1-phosphate (S1P), a potent lipid-signaling molecule that activates G-protein-coupled receptors and has a wide variety of functions [7], was more abundant in plasma from subjects with PTSD (p<0.046; q=0.38). S1P regulates cell proliferation, survival and differentiation of neuronal cells and muscle cells [8] regulation of cell-cell and cell-matrix adhesion [7], immune cell trafficking of B-cells and T-cells [9], induction of cyclooxygenase activity [10], regulation of angiogenesis, vascular stability, and vascular permeability [7, 11]. The observed difference in plasma concentrations of S1P thus may affect several signaling pathways in several cell types.

**Bootstrapping and Power Analysis in PTSD Metabolomics Dataset**

**1. Prediction of Type 1 errors in the 52-52 discovery cohort**

Using the q-values from Table 2, we can predict that less than 37.8% of significant 52-52 findings will be False Positives due to multiple comparisons. But the 31-31 dataset is unable to validate 15/23, or 65% of the findings. This is much higher than the false positive rate we would expect. This is most likely due to either (1) lack of power in the test dataset or (2) population differences between the 52- 52 and 31-31 datasets.

**2. Estimating power**

Power in the test dataset can be estimated by using the effect sizes from the 52-52 cohort as estimates of the population effect sizes. The metabolite effect sizes (Cohen’s d) from the significant 52-52 findings range from 0.275-0.963. Using a t-test scenario, assuming, for simplicity, that the covariates are relatively unimportant, the power in a test dataset of sample size, N=62 can be computed. Our estimate of the power (the probability of finding an effect that is there), ranges from 18% to 96%, with most metabolites at less than 50% power. This is likely why many of the findings in the 31-31 cohort were unable to be validated. The current sample size does not provide enough power to see the potential signals.

**3. Potential signal differences between the 52-52 and 31-31 groups**

The other possible explanation for the small number of validated findings in the test group is that there are signal differences between the discovery and test groups. Performing resampling to shuffle the 52-52 and 31-31 together should help overcome this problem.

The following steps for were performed for this resampling analysis:

1. Resample the data 1000 times into discovery and test groups. Because of the surprisingly low power in the 31-31 dataset, we made these groups equal, with 83 subjects in each.
2. For each of the 1000 groupings, attempt to "discover" significant metabolites in the first group, and then validate those significant discoveries in the second group.
3. For each metabolite, track how many of the 1000 groupings resulted in significance in both discovery and validation.

The results from this analysis are shown in S1 Fig A, which shows a barplot of the most frequent metabolites. The bar height indicates the fraction of success in both discovery and validation. After the metabolite "gammaglutamylleucine", which was successfully discovered and validated 5 out of 1000 times, all 225 other metabolites were validated less than 5 times. Metabolites discovered and validated more than 4 out of 100 times (0.04%) are significant at the p=0.05 level.


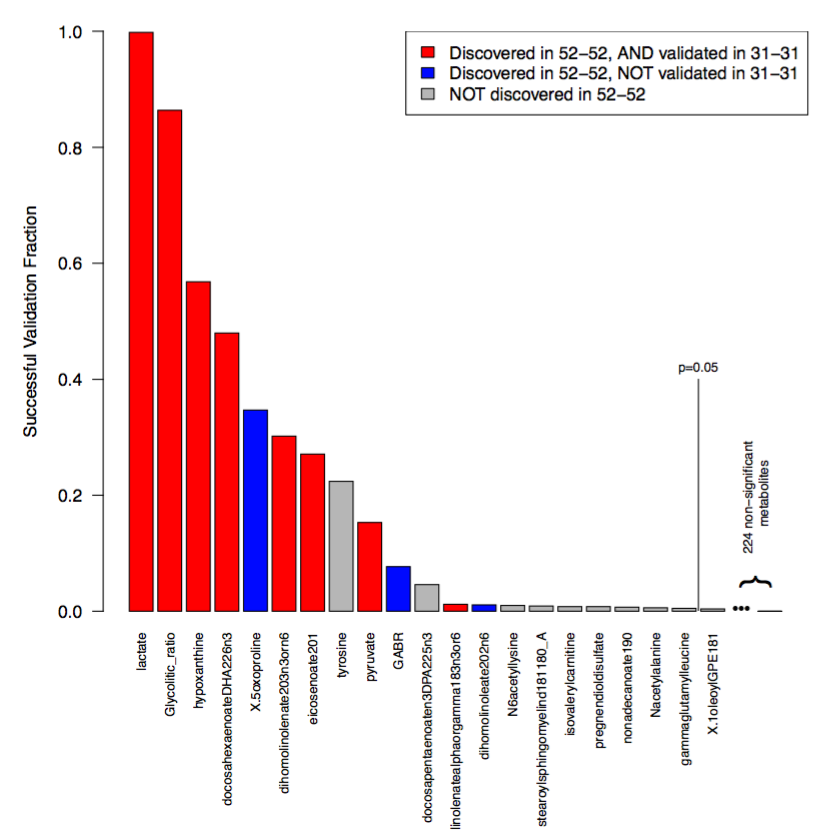


**S1 File Fig A. Barplot of metabolite validation success fraction over 1000 permutations of discovery and test groups**.

These analyses suggest that our initial discovery cohort correctly identified the strongest metabolite signatures that were different between PTSD positive and negative subjects, and that there was nothing inherently distinct about the subjects in the discovery group. This analysis further suggests that placing other subjects in this discovery or test groups would not have identified additional metabolites that were significantly different between PTSD positive and negative subjects with the exception of tyrosine, docosapentaenoic acid (another omega 3 essential fatty acid), n-acetyl lysine, steroylsphoingomyelin, isovalerylcarnitine (another acyl carnitine), pregnendioldisulfate, nonadecanoate (another fatty acid), n-acetyl alanine and gamma-glutamyl leucine.

**Discussion of Supporting Results**

**Carnitine regulation of fatty acids**

Concentrations of several acylcarnitines were increased in PTSD. Carnitines, conjugated to acetyl CoA, transport long chain fatty acids into mitochondria for β-oxidation, and in the process, release acetyl CoA used in the TCA cycle. Both free carnitines and acylcarnitines regulate fatty acid metabolism, ketosis, and buffer the ratio of acyl-CoA to free CoA. Carnitines may also have roles in neuroprotection [12]. Brain acylcarnitines participate in synthesizing lipids, altering and stabilizing membrane composition, modulating genes and proteins, improving mitochondrial function, increasing antioxidant activity, and enhancing cholinergic neurotransmission. When given to dogs, acyl carnitines improved neurological outcome in models of brain injury, potentially as a result of normalizing brain energy metabolites [13]. Recent studies have indicated that lower plasma concentrations of acylcarnities may predict the development of cognitive impairment in elderly subjects[14]. By contrast, in studies in rats, medium chain acyl carnitines (hexanoylcarnitine, octanoylcarnitine, decanoylcarnitine – the same acyl carnitines we found to be elevated in our PTSD subjects) and cis-4-decenoylcarnitine, but not L-carnitine itself, significantly induced lipid peroxidation and protein oxidative damage, increased protein oxidation, and decreased levels of glutathione, a potent antioxidant [15]. These effects were mediated by increased concentrations of reactive oxygen species. Thus, it is unclear how acylcarnitines can be both neuroprotective *and* increase reactive oxygen species.

**Urea Cycle**

Arginine, a component of the urea cycle, was lower in subjects with PTSD vs. controls. Arginine participates in carbon-nitrogen balance, eliminating nitrogenous waste, and is the substrate from which endothelial nitric oxide (NO) synthase (eNOS) produces NO; thus reduced arginine concentrations may reduce NO. Because NO promotes vascular dilation, reduced NO may have deleterious effects on the cardiovascular system. Moreover, eNOS produces superoxide when depleted of arginine and tetrahydro-L-biopterinby uncoupling electron flow from NO production. Superoxide production may result in additional mitochondrial dysfunction. High glucose levels, as seen in the PTSD subjects, impair eNOS activity via increased *O*-linked glycosylation, and may result in further uncoupling of eNOS and increased generation of superoxide.

In a group of >1000 subjects, low ratios of arginine to (citrulline + ornithine) (the Global Arginine Bioavailability Ratio, GABR) were associated with coronary artery disease and major adverse cardiovascular events, including myocardial infarction, stroke, and death, three years later [16]. As we showed previously [17], our discovery group PTSD subjects had a lower GABR score than our control subjects (p<0.009). Since lower GABR ratios are associated with increased coronary artery disease, these data suggest that biological mechanisms involving GABR may heighten this increased risk for cardiovascular disease in PTSD subjects.

**References**

1. Evans AM, DeHaven CD, Barrett T, Mitchell M, Milgram E. Integrated, nontargeted ultrahigh performance liquid chromatography/electrospray ionization tandem mass spectrometry platform for the identification and relative quantification of the small-molecule complement of biological systems. Analytical chemistry. 2009;81(16):6656-67. Epub 2009/07/25. doi: 10.1021/ac901536h. PubMed PMID: 19624122.

2. Ohta T, Masutomi N, Tsutsui N, Sakairi T, Mitchell M, Milburn MV, et al. Untargeted metabolomic profiling as an evaluative tool of fenofibrate-induced toxicology in Fischer 344 male rats. Toxicologic pathology. 2009;37(4):521-35. Epub 2009/05/22. doi: 10.1177/0192623309336152. PubMed PMID: 19458390.

3. Dehaven CD, Evans AM, Dai H, Lawton KA. Organization of GC/MS and LC/MS metabolomics data into chemical libraries. Journal of cheminformatics. 2010;2(1):9. Epub 2010/10/20. doi: 10.1186/1758-2946-2-9. PubMed PMID: 20955607; PubMed Central PMCID: PMC2984397.

4. Ishii N, Ikenaga H, Carmines PK, Aoki Y, Ogawa Z, Saruta T, et al. High glucose augments arginase activity and nitric oxide production in the renal cortex. Metabolism: clinical and experimental. 2004;53(7):868-74. Epub 2004/07/16. PubMed PMID: 15254879.

5. Dillon EL, Knabe DA, Wu G. Lactate inhibits citrulline and arginine synthesis from proline in pig enterocytes. The American journal of physiology. 1999;276(5 Pt 1):G1079-86. PubMed PMID: 10329997.

6. Jang C, Oh SF, Wada S, Rowe GC, Liu L, Chan MC, et al. A branched-chain amino acid metabolite drives vascular fatty acid transport and causes insulin resistance. Nature medicine. 2016;22(4):421-6. doi: 10.1038/nm.4057. PubMed PMID: 26950361; PubMed Central PMCID: PMCPMC4949205.

7. Mendelson K, Evans T, Hla T. Sphingosine 1-phosphate signalling. Development. 2014;141(1):5-9. Epub 2013/12/19. doi: 10.1242/dev.094805. PubMed PMID: 24346695; PubMed Central PMCID: PMC3865745.

8. Donati C, Cencetti F, Bruni P. Sphingosine 1-phosphate axis: a new leader actor in skeletal muscle biology. Frontiers in physiology. 2013;4:338. Epub 2013/12/11. doi: 10.3389/fphys.2013.00338. PubMed PMID: 24324439; PubMed Central PMCID: PMC3839259.

9. Garris CS, Blaho VA, Hla T, Han MH. Sphingosine-1-phosphate receptor 1 signalling in T cells: trafficking and beyond. Immunology. 2014;142(3):347-53. Epub 2014/03/07. doi: 10.1111/imm.12272. PubMed PMID: 24597601; PubMed Central PMCID: PMC4080950.

10. Nakamura H, Murayama T. Role of sphingolipids in arachidonic acid metabolism. Journal of pharmacological sciences. 2014;124(3):307-12. Epub 2014/03/07. PubMed PMID: 24599139.

11. Curry FR, Adamson RH. Tonic regulation of vascular permeability. Acta Physiol (Oxf). 2013;207(4):628-49. Epub 2013/02/05. doi: 10.1111/apha.12076. PubMed PMID: 23374222; PubMed Central PMCID: PMC4054936.

12. Jones LL, McDonald DA, Borum PR. Acylcarnitines: role in brain. Progress in lipid research. 2010;49(1):61-75. Epub 2009/09/02. doi: 10.1016/j.plipres.2009.08.004. PubMed PMID: 19720082.

13. Rosenthal RE, Williams R, Bogaert YE, Getson PR, Fiskum G. Prevention of postischemic canine neurological injury through potentiation of brain energy metabolism by acetyl-L-carnitine. Stroke; a journal of cerebral circulation. 1992;23(9):1312-7; discussion 7-8. Epub 1992/09/01. PubMed PMID: 1519288.

14. Mapstone M, Cheema AK, Fiandaca MS, Zhong X, Mhyre TR, Macarthur LH, et al. Plasma phospholipids identify antecedent memory impairment in older adults. Nature medicine. 2014;20(4):415-8. doi: 10.1038/nm.3466. PubMed PMID: 24608097; PubMed Central PMCID: PMCPMC5360460.

15. Tonin AM, Grings M, Knebel LA, Zanatta A, Moura AP, Ribeiro CA, et al. Disruption of redox homeostasis in cerebral cortex of developing rats by acylcarnitines accumulating in medium-chain acyl-CoA dehydrogenase deficiency. International journal of developmental neuroscience : the official journal of the International Society for Developmental Neuroscience. 2012;30(5):383-90. Epub 2012/04/05. doi: 10.1016/j.ijdevneu.2012.03.238. PubMed PMID: 22472139.

16. Tang WH, Wang Z, Cho L, Brennan DM, Hazen SL. Diminished global arginine bioavailability and increased arginine catabolism as metabolic profile of increased cardiovascular risk. Journal of the American College of Cardiology. 2009;53(22):2061-7. Epub 2009/05/30. doi: 10.1016/j.jacc.2009.02.036. PubMed PMID: 19477356; PubMed Central PMCID: PMC2755213.

17. Bersani FS, Wolkowitz OM, Lindqvist D, Yehuda R, Flory J, Bierer LM, et al. Global arginine bioavailability, a marker of nitric oxide synthetic capacity, is decreased in PTSD and correlated with symptom severity and markers of inflammation. Brain, behavior, and immunity. 2016;52:153-60. doi: 10.1016/j.bbi.2015.10.015. PubMed PMID: 26515034.
